# Mutant human torsinA, responsible for early-onset dystonia, dominantly suppresses GTPCH expression, dopamine levels and locomotion in *Drosophila melanogaster*

**DOI:** 10.1242/bio.201411080

**Published:** 2015-04-17

**Authors:** Noriko Wakabayashi-Ito, Rami R. Ajjuri, Benjamin W. Henderson, Olugbenga M. Doherty, Xandra O. Breakefield, Janis M. O'Donnell, Naoto Ito

**Affiliations:** 1 Department of Neurology, Massachusetts General Hospital and Program in Neuroscience, Harvard Medical School, Boston, MA 02129, USA; 2 Department of Biological Sciences, University of Alabama, Tuscaloosa, AL 35487, USA

**Keywords:** Dystonia, *Drosophila*, GTP cyclohydrolase, TorsinA, Movement disorder

## Abstract

Dystonia represents the third most common movement disorder in humans with over 20 genetic loci identified. *TOR1A (DYT1)*, the gene responsible for the most common primary hereditary dystonia, encodes torsinA, an AAA ATPase family protein. Most cases of DYT1 dystonia are caused by a 3 bp (ΔGAG) deletion that results in the loss of a glutamic acid residue (ΔE302/303) in the carboxyl terminal region of torsinA. This torsinAΔE mutant protein has been speculated to act in a dominant-negative manner to decrease activity of wild type torsinA. *Drosophila melanogaster* has a single torsin-related gene, *dtorsin*. Null mutants of *dtorsin* exhibited locomotion defects in third instar larvae. Levels of dopamine and GTP cyclohydrolase (GTPCH) proteins were severely reduced in *dtorsin*-null brains. Further, the locomotion defect was rescued by the expression of human torsinA or feeding with dopamine.

Here, we demonstrate that human torsinAΔE dominantly inhibited locomotion in larvae and adults when expressed in neurons using a pan-neuronal promoter Elav. Dopamine and tetrahydrobiopterin (BH_4_) levels were significantly reduced in larval brains and the expression level of GTPCH protein was severely impaired in adult and larval brains. When human torsinA and torsinAΔE were co-expressed in neurons in *dtorsin*-null larvae and adults, the locomotion rates and the expression levels of GTPCH protein were severely reduced. These results support the hypothesis that torsinAΔE inhibits wild type torsinA activity. Similarly, neuronal expression of a *Drosophila* DtorsinΔE equivalent mutation dominantly inhibited larval locomotion and GTPCH protein expression. These results indicate that both torsinAΔE and DtorsinΔE act in a dominant-negative manner. We also demonstrate that Dtorsin regulates GTPCH expression at the post-transcriptional level. This *Drosophila* model of DYT1 dystonia provides an important tool for studying the differences in the molecular function between the wild type and the mutant torsin proteins.

## Introduction

Dystonia is the third most common movement disorder in humans, after essential tremor and Parkinson's disease (Defazio, 2010). Dystonia comprises a group of movement disorders that are characterized by involuntary movements and abnormal postures. It is a complex disease involving at least 20 genetic loci in humans (Tarsy and Simon, 2006; Breakefield et al., 2008; Brüggemann and Klein, 2010).

One of the loci, *TOR1A/DYT1*, is responsible for most cases of early-onset dystonia and has been the most studied form of dystonia (Breakefield et al., 2001; Atai et al., 2012; Bragg et al., 2011). It is an autosomal dominant syndrome with onset between 5 to 28 years of age and low penetrance. The *TOR1A* gene encodes torsinA, a 332 amino acid protein from the AAA ATPase family. The torsinA protein is widely expressed in the body and is localized within the lumen of the endoplasmic reticulum and the nuclear envelope (Breakefield et al., 2008), but its function is still under study. A 3-bp (ΔGAG) deletion that removes one of a pair of glutamic acid residues (ΔE302/E303) in the carboxyl terminal region of torsinA causes the autosomal dominant dystonia phenotype (Breakefield et al., 2008; Bragg et al., 2011). TorsinA displays LAP1 and LULL1-dependent ATPase activity, while the torsinAΔE protein is defective in this activation (Zhao et al., 2013). The torsinAΔE (ΔE302/303) mutant protein has been speculated to act in a dominant-negative manner, so that the wild type function is reduced but not eliminated in the cells expressing both torsinA and torsinAΔE, although this has never been clearly demonstrated (Breakefield et al., 2001; Breakefield et al., 2008).

Most AAA ATPase proteins form oligomeric complexes and use energy from ATP hydrolysis to regulate protein folding, membrane trafficking, and vesicle fusion (Neuwald et al., 1999; Vale, 2000; Hanson and Whiteheart, 2005; Zhao et al., 2013). Although torsinA is widely expressed in human tissue, it is considered to have a critical role in the central nervous system, where it is present in neurons at high levels during development and in adult life (Augood et al., 2003; Xiao et al., 2004; Vasudevan et al., 2006). In homozygous torsinA-knock-out mice, abnormal nuclear membrane morphology was observed in neurons, suggesting a functional role of torsinA in maintaining the normal structure of the nuclear envelope in the central nervous system (Goodchild et al., 2005). TorsinA has been shown to interact with nesprins, which are anchored in the outer nuclear envelope and form bridges to the cytoskeleton (Nery et al., 2008; Jungwirth et al., 2011; Atai et al., 2012), suggesting an important functional role of torsinA at the nuclear envelope, including nuclear polarization during cell migration (Nery et al., 2008). Recent studies also implicate torsinA in egress of Herpes simplex virus capsids (Maric et al., 2011) and large ribonucleoprotein particles (Jokhi et al., 2013) out from the nucleus into cytoplasm.

The fruit fly, *Drosophila melanogaster*, provides an excellent model system to study functions of human disease genes and has contributed to better understanding of many human diseases (Bellen et al., 2010). *Drosophila* has a single *TOR1A*-related gene, *dtorsin* (*Torsin*), at position 4C11 on the X chromosome (Ozelius et al., 1999; Breakefield et al., 2001; Wakabayashi-Ito et al., 2011). The *dtorsin*-encoded protein, Dtorsin, comprises 339 amino acids with 31.9% identity to human torsinA and also displays the characteristic features of the AAA ATPase gene family members (supplementary material Fig. S1) (Ozelius et al., 1999). We recently isolated *dtorsin*-null mutants and showed that hemizygous mutant third instar male larvae exhibited locomotion defects that were rescued by feeding dopamine (Wakabayashi-Ito et al., 2011). The *dtorsin*-null mutation was semi-lethal at the pupal stage with only less than 1% reaching adult stage. The *dtorsin* mutant exhibited a very strong genetic interaction with *Pu* (*Punch*: GTP cyclohydrolase: GTPCH), the ortholog of the human gene underlying dopa-responsive DYT5a dystonia (*GCH1*) (Segawa, 2009). Moreover, biochemical analysis revealed a severe reduction of GTPCH protein and activity in *dtorsin*-null adults and larvae, as well as marked reduction in tetrahydrobiopterin (BH_4_), the terminal product of the GTPCH pathway. In contrast, levels of tyrosine hydroxylase (TH) protein, which catalyzes the rate limiting step in dopamine production, were not affected, although dopamine pools were reduced (Wakabayashi-Ito et al., 2011). Since GTPCH is rate limiting for the synthesis of BH_4_, and BH_4_ is required by TH as a rate-limiting cofactor for dopamine synthesis in flies as in mammals (Krishnakumar et al., 2000), these data suggested that *dtorsin* plays a novel role in dopamine metabolism as a positive-regulator of GTPCH protein levels in *Drosophila*. Moreover, the wild type human torsinA cDNA expressed with the pan-neuronal promoter elavGAL4 rescued *dtorsin*-null male larval mobility with marked significance. These results demonstrated that the function of torsin in regulating larval locomotion is conserved between the fly and the human proteins (Wakabayashi-Ito et al., 2011). However, the fly *dtorsin*-null mutant is not an authentic DYT1 disease model system, since the *dtorsin*-null mutant line does not express any functional Dtorsin protein, while mutated torsinA protein is expressed together with normal torsinA in the DYT1 patients (Breakefield et al., 2001).

To investigate the molecular mechanism underlying the human disease caused by mutated torsinA protein using the fly system, we expressed human wild type torsinA and/or torsinAΔE cDNA using the pan-neuronal GAL4 driver, elavGAL4, in fly brains. We report here that expression of the human mutant form caused larval and adult locomotion defects, and severe reduction of GTPCH protein, dopamine, and BH_4_ levels in larval brains and adult heads. Moreover, co-expression of human torsinAΔE and the wild type human torsinA in *dtorsin*-null males resulted in similar larval/adult locomotion and neurochemical defects, suggesting that the human torsinAΔE exerts dominant-negative effects on human wild type torsinA protein in *Drosophila* neurons, as in human tissues. Furthermore, a comparable mutation in the *Drosophila* gene, *dtorsin*Δ*E* also had a dominant-negative effect on larval locomotion and GTPCH protein level, as did human torsinAΔE. Finally, we report that the relative amount of GTPCH RNA was similar in wild type and *dtorsin*-null adult male heads, suggesting that GTPCH protein levels depend on wild type dtorsin-activity at the post-transcriptional level. Our findings establish conclusively that torsinAΔE dominantly inhibits the normal function of torsinA and Dtorsin including the regulation of GTPCH expression. These results demonstrate that *Drosophila* provides a powerful system for studying the molecular abnormalities caused by the torsinAΔE mutation.

## Results

### Human torsinAΔE dominantly inhibits larval locomotion

**Fig. 1.**
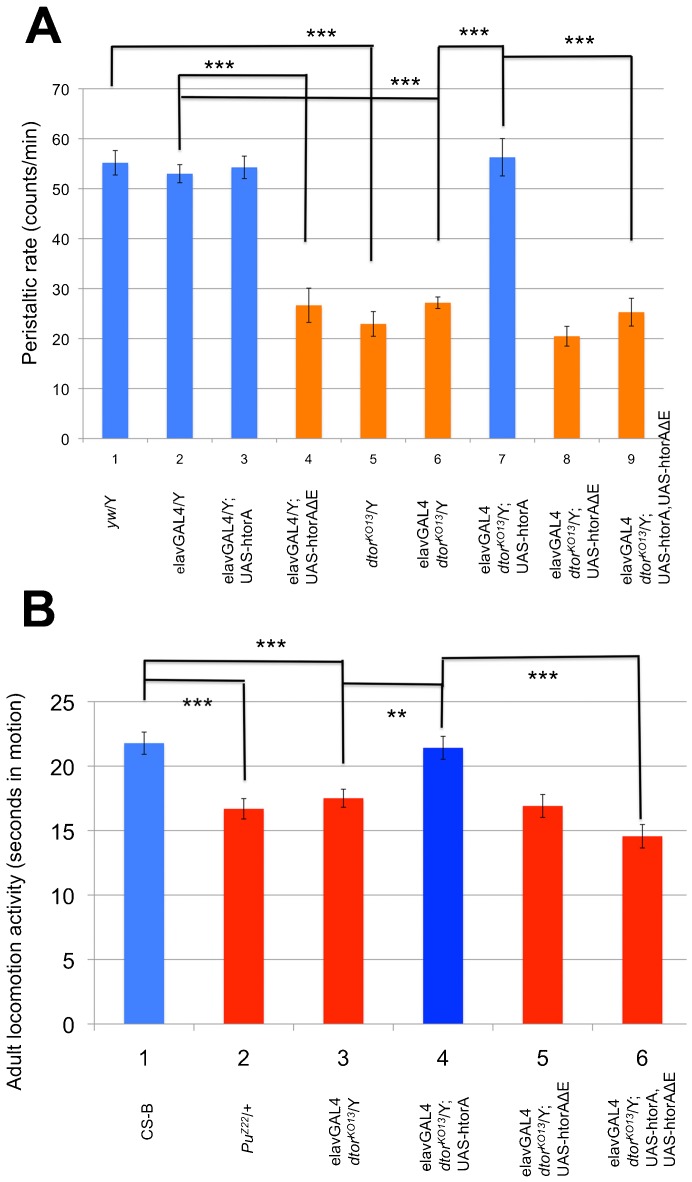
Neuronal expression of human torsinAΔE has a dominant-negative effect on larval and adult locomotion. (A) Peristaltic frequencies were counted for the wandering stage third instar larvae of the genotype: (1) *y w*/Y (wild type) male (n = 15), (2) *w elavGAL4*/Y (wild type) male (n = 9), (3) *w elavGAL4*/Y; UAS-htorsinA/+ male (n = 15), (4) *w elavGAL4*/Y; UAS-htorsinAΔE/+ male (n = 9), (5) *y w dtorsin^KO13^*/Y (*dtorsin*-null) male (n = 14), (6) *w elavGAL4 dtorsin^KO13^*/Y (*dtorsin*-null) male (n = 39), (7) *w elavGAL4 dtorsin^KO13^*/Y; UAS-htorsinA/+ male (n = 14), (8) *w elavGAL4 dtorsin^KO13^*/Y; UAS-htorsinAΔE/+ male (n = 21), (9) *w elavGAL4 dtorsin^KO13^*/Y; UAS-htorsinA, UAS-htorsinAΔE/+ male (n = 14). Results are expressed as the mean±S.E.M. ***p<0.0001. (B) Adult locomotion activities were measured for the adults of the genotype: (1) Canton S-B (wild type) male (n = 47), (2) *Pu^Z22^*/+ (*Pu* null mutation) male (n = 44), (3) *w elavGAL4 dtorsin^KO13^*/Y (*dtorsin*-null) male (n = 64), (4) *w elavGAL4 dtorsin^KO13^*/Y; UAS-htorsinA/+ male (n = 77), (5) *w elavGAL4 dtorsin^KO13^*/Y; UAS-htorsinAΔE/+ male (n = 69), (6) *w elavGAL4 dtorsin^KO13^*/Y; UAS-htorsinA, UAS-htorsinAΔE/+ male (n = 63). Results are expressed as the mean±S.E.M. ***p<0.001, **p<0.05.

In the previous study, we analyzed the peristaltic frequency of third instar larvae to quantify the difference in locomotion between wild type and mutant. The wild type third instar larvae show approximately 55 muscle contraction cycles per minute when placed on 0.7% agarose plates at room temperature. These peristaltic rates are relatively easy to monitor and provide a sensitive and reliable way of quantifying larval locomotion (Song et al., 2007; Wakabayashi-Ito et al., 2011). Males of the null mutant, *dtorsin^KO13^*, exhibit approximately a ∼50% decrease in peristaltic rates, 22.9±2.5 (n = 28, p<0.0001) (Fig. 1A, column 5), compared to wild type (55.2±2.5, n = 15) (Fig. 1A, column 1). As previously observed, the wild type human torsinA cDNA expressed with the pan-neuronal driver elavGAL4 rescued *dtorsin^KO13^* male larval mobility to a very significant level (56.3±3.7, n = 14, p<0.0001) (Fig. 1A, column 7) (Wakabayashi-Ito et al., 2011), compared to *dtorsin^KO13^* male larvae with the elavGAL4 transgene (27.2±1.1, n = 39) (Fig. 1A, column 6). By way of controls, the pan-neuronal expression of the wild type human torsinA cDNA in wild type flies had no effect on larval mobility (54.3±2.3, n = 15, p = 0.7) (Fig. 1A, column 3), compared to male larvae with elavGAL4 transgene alone (53.0±1.8, n = 9) (Fig. 1A, column 2). Similarly, the presence/absence of the elavGAL4 transgene had no effect on mobility in wild type (Fig. 1A, columns 1, 2) and *dtorsin^KO13^* larvae (Fig. 1A, columns 5,6).

To examine the effect of mutated human torsinAΔE protein in flies, we expressed human torsinAΔE cDNA with the pan-neuronal elavGAL4 driver in wild type males (*w dtorsin^+^*). ElavGAL4/UAS-htorsinAΔE males exhibited a severe locomotion deficit, approaching that of the *dtorsin*-null mutant (26.7±3.4, n = 9, p<0.0001) (Fig. 1A, column 4, compared to column 2). This result demonstrates that pan-neuronal expression of human torsinAΔE protein has a negative effect on larval locomotion, similar to the *dtorsin*-null state in flies, and that it interferes with the function of endogenous Dtorsin.

While pan-neuronal expression of human wild type torsinA could rescue the locomotion deficit phenotype of *dtorsin^KO13^* males (Fig. 1A, columns 6,7), human torsinAΔE was unable to do so (20.5±2.0, n = 21) (Fig. 1A, column 8). To determine whether the human torsinAΔE could inhibit wild type human torsinA, we co-expressed the human torsinAΔE cDNA with the wild type human torsinA cDNA in *dtorsin^KO13^* male using the same elavGAL4 driver. Co-expression of human wild type torsinA and human torsinAΔE resulted in a significant inhibition of mobility (25.3±2.8, n = 14, p<0.0001) (Fig. 1A, column 9), compared to the rescue by human torsinA alone (Fig. 1A, column 7).

### Human torsinAΔE dominantly inhibits adult locomotion

We have also analyzed the locomotion activities in the adult stage to examine whether they were similarly affected by the neuronal expression of human torsinAΔE. Adult flies, aged 3–5 days after eclosion, were placed in vials, subjected to a gentle mechanical disturbance, and then locomotion activities were quantified as the number of seconds each fly spent in motion during a 45 second period (Carbone et al., 2006). Adult wild type male flies (Canton S-B) spent approximately 21.8±0.9 seconds in motion (n = 47) (Fig. 1B, column 1). Adult male flies that were heterozygous for a lethal *Punch* (GTPCH gene) null mutation, *Pu^Z22^*/+ (Mackay et al., 1985), exhibit a significant reduction of locomotion activities with 16.7±0.8 seconds spent in motion (n = 44, p<0.0001) (Fig. 1B, column 2), compared to wild type (Fig. 1B, column 1). Similarly, adult males of the null mutant, *dtorsin^KO13^*, exhibit a significant reduction of adult locomotion activities, (17.5±0.7 seconds, n = 64, p = 0.0002) (Fig. 1B, column 3), as observed in the third instar larvae. The wild type human torsinA cDNA expressed with the pan-neuronal driver elavGAL4 strongly rescued *dtorsin^KO13^* male adult locomotion activities (21.4±0.9 seconds, n = 77) (Fig. 1B, column 4), compared to *dtorsin^KO13^* adult males (Fig. 1B, column 3) (p = 0.01). The mutant form of torsinAΔE was unable to rescue the adult locomotion defect (16.9±0.9 seconds, n = 69) (Fig. 1B, column 5) (p = 0.603, compared to column 3). Co-expression of the human torsinAΔE with the wild type human torsinA cDNA in *dtorsin^KO13^* adult male resulted in a significant reduction of locomotion activities (14.6±0.9, n = 63) (Fig. 1B, column 6) compared to the rescue by human torsinA alone (Fig. 1B, column 4) (p<0.0001). These results demonstrate that adult locomotion activities in flies are dominantly inhibited by the neuronal expression of the mutant form of human torsinA.

### Human torsinAΔE dominantly suppresses GTPCH expression

**Fig. 2.**
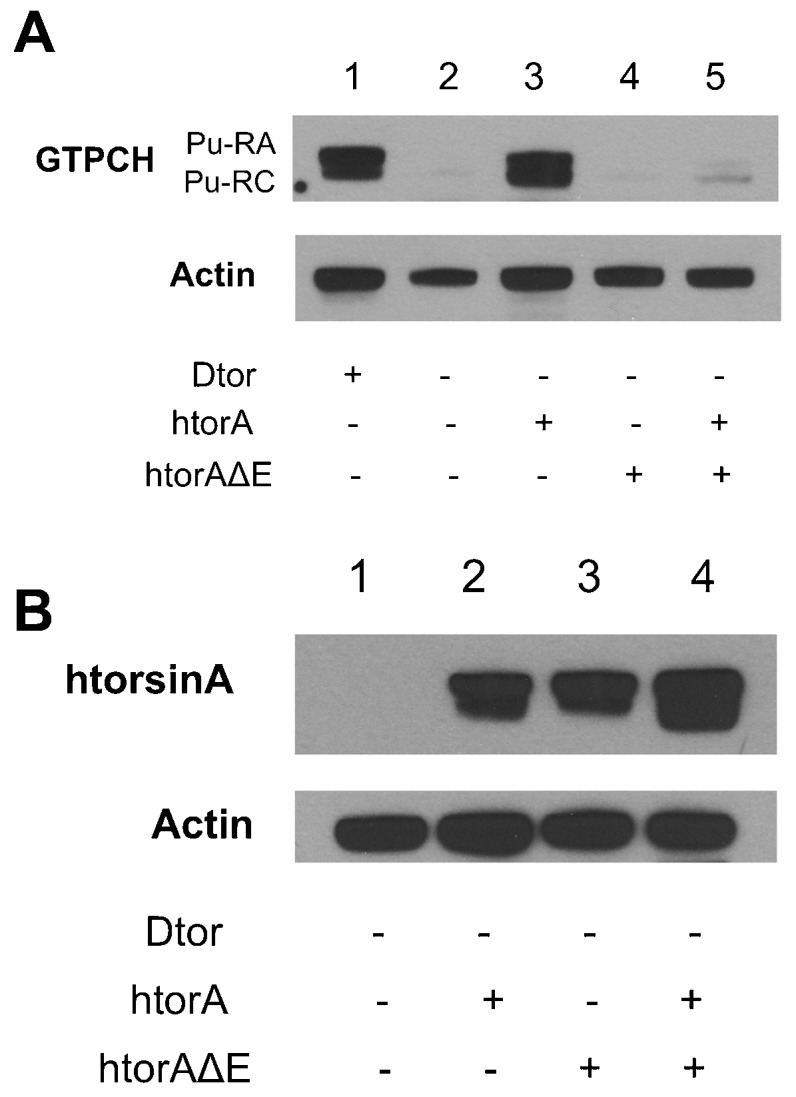
Neuronal expression of human torsinAΔE has a dominant-negative effect on GTPCH protein levels in adult brains. (A) Adult head extracts were analyzed by western blots. The membrane was probed with rabbit anti-GTPCH A/C (upper panel) and reprobed with rabbit anti-actin (lower panel). The genotypes are: (1) *y w* (wild type) males, (2) *w elavGAL4 dtorsin^KO13^*/Y (*dtorsin*-null) males, (3) *w elavGAL4 dtorsin^KO13^*/Y; UAS-htorsinA/+ males, (4) *w elavGAL4 dtorsin^KO13^*/Y; UAS-htorsinAΔE/+ males, (5) *w elavGAL4 dtorsin^KO13^*/Y; UAS-htorsinA, UAS-htorsinAΔE/+ males. The locations of GTPCH (Pu-RA: 45 kDa, Pu-RC: 43 kDa) and actin (42 kDa) are indicated. Thirty µg of proteins were loaded in each lane. (B) Adult head extracts were analyzed by western blots. The membrane was probed with rabbit anti-human torsinA and reprobed with rabbit anti-actin antibodies. The genotypes are: (1) *w elavGAL4 dtorsin^KO13^*/Y (*dtorsin*-null) males, (2) *w elavGAL4 dtorsin^KO13^*/Y; UAS-htorsinA/+ males, (3) *w elavGAL4 dtorsin^KO13^*/Y; UAS-htorsinAΔE/+ males, (4) *w elavGAL4 dtorsin^KO13^*/Y; UAS-htorsinA/+; UAS-htorsinAΔE/+ males. The locations of human torsinA (37 kDa) (double bands) and actin (42 kDa) are indicated. Thirty µg of proteins were loaded in each lane.

We have previously shown, and confirm here, that *dtorsin^KO13^* males have a severe reduction of both the 45 kD (Pu-RA) and 43 kD (Pu-RC) isoforms of GTPCH protein in adult brains (Fig. 2A, lane 1 and 2; supplementary material Fig. S3A, columns 1, 2) (Wakabayashi-Ito et al., 2011). Males heterozygous for the embryo-lethal *Pu* null mutation, *Pu^Z22^*/+ (Mackay et al., 1985), had a severe reduction of both Pu-RA and Pu-RC isoforms (supplementary material Fig. S2, lanes 1, 2), confirming that these two polypeptides are encoded by the GTPCH gene. To investigate whether human torsinAΔE has a similar effect on GTPCH, we prepared extracts from heads of *dtorsin^KO13^* adult males expressing human torsinAΔE in neurons, *dtorsin^KO13^* adult males expressing wild type torsinA in neurons, and *dtorsin^KO13^* adult males expressing both human torsinA and human torsinAΔE in neurons, and compared GTPCH protein levels by western blot analysis (Fig. 2A, lanes 3–5; supplementary material Fig. S3A, columns 3–5). Pan-neuronal expression of human torsinAΔE in *dtorsin^KO13^* adult males, confirmed by immunoblotting using an antibody specific to human torsinA (Bragg et al., 2004) (Fig. 2B, lane 3), revealed that the mutant human torsinA protein was unable to rescue GTPCH protein levels when expressed alone in *dtorsin^KO13^* adult males (Fig. 2A, lane 4 compared to lane 2). In contrast, neuronal expression of wild type human torsinA alone (Fig. 2B, lane 2) strongly rescued both isoforms of GTPCH in *dtorsin*-null males (compare Fig. 2A, lanes 2 and 3). Severe reduction of GTPCH was observed in *dtorsin^KO13^* adult males expressing human torsinA and human torsinAΔE together (Fig. 2A, lane 5 compared to lane 3), even though the expression of torsinAΔE with the wild type form does not diminish the total level of human torsinA expressed in fly neurons (Fig. 2B, lane 4; supplementary material Fig. S3B).

**Fig. 3.**
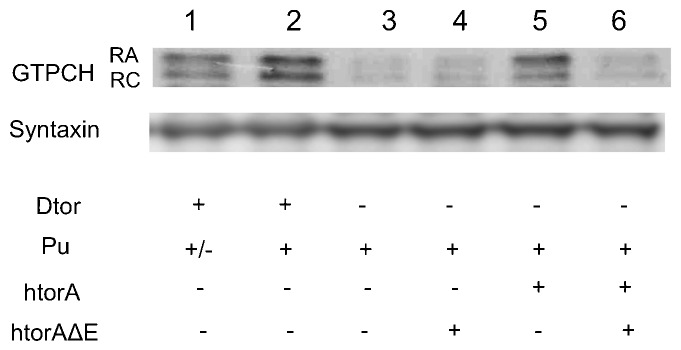
Neuronal expression of human torsinAΔE has a dominant-negative effect on GTPCH protein levels in larval brains. Larval brain extracts were analyzed by western blots. The membrane was probed with rabbit anti-GTPCH (upper panel) and reprobed with mouse anti-syntaxin (lower panel). The genotypes are: (1) *Pu^Z22^*/+ (*Pu* null mutation) males, (2) Canton S-B (wild type) males, (3) *y w dtorsin^KO13^*/Y (*dtorsin*-null) males, (4) *w elavGAL4 dtorsin^KO13^*/Y; UAS-htorsinAΔE/+ males, (5) *w elavGAL4 dtorsin^KO13^*/Y; UAS-htorsinA/+ males, (6) *w elavGAL4 dtorsin^KO13^*/Y; UAS-htorsinA, UAS-htorsinAΔE/+ males. Two isoforms of GTPCH (Pu-RA: 45 kDa, Pu-RC: 43 kDa) are expressed in the brain of wild type flies. Both were reduced in *Pu^z22^*/+ heterozygotes, indicating that both of these proteins are encoded by the *Pu* gene (Mackay et al., 1985). In addition, both isoforms are severely reduced in the *dtorsin^KO13^* null hemizygous brain extracts. The htorsinAΔE transgene is unable to recue GTPCH expression in the *dtorsin*-null background, while the wild type htorsinA transgene strongly rescued the both isoforms of GTPCH in larval brains. The presence of the htorsinAΔE transgene prevents wild type htorsinA rescue of GTPCH in the *dtorsin*-null background. Anti-syntaxin was employed as a loading control. Twenty µg of total brain proteins were loaded in each lane.

We obtained comparable results using brain extracts from third instar larvae of the corresponding genotypes (Fig. 3; supplementary material Figs S4, S5) as those of adult head extracts (Fig. 2; supplementary material Fig. S3). That is, the htorsinAΔE transgene fails to rescue either isoform of brain GTPCH, both of which are affected by complete knockout of the *dtorsin* gene (Fig. 3, lane 3 and 4; supplementary material Fig. S4, columns 3, 4). Expression of wild type htorsinA (Fig. 3, lane 5; supplementary material Fig. S4, column 5) rescues expression of both isoforms (Pu-RA and Pu-RC) of GTPCH expression with Pu-RA rescue slightly more effectively than Pu-RC. The basis for this slight difference is unclear at this time. Nevertheless, these results confirm that wild type human torsinA is capable of rescuing neuronal expression of *Drosophila* GTPCH and demonstrate that the human torsinAΔE, when co-expressed with the wild type human transgene, dominantly suppresses GTPCH protein levels in both larval and adult brains without negatively affecting the expression of wild type human torsinA.

### Human torsinAΔE dominantly reduces BH_4_ and dopamine level

**Fig. 4.**
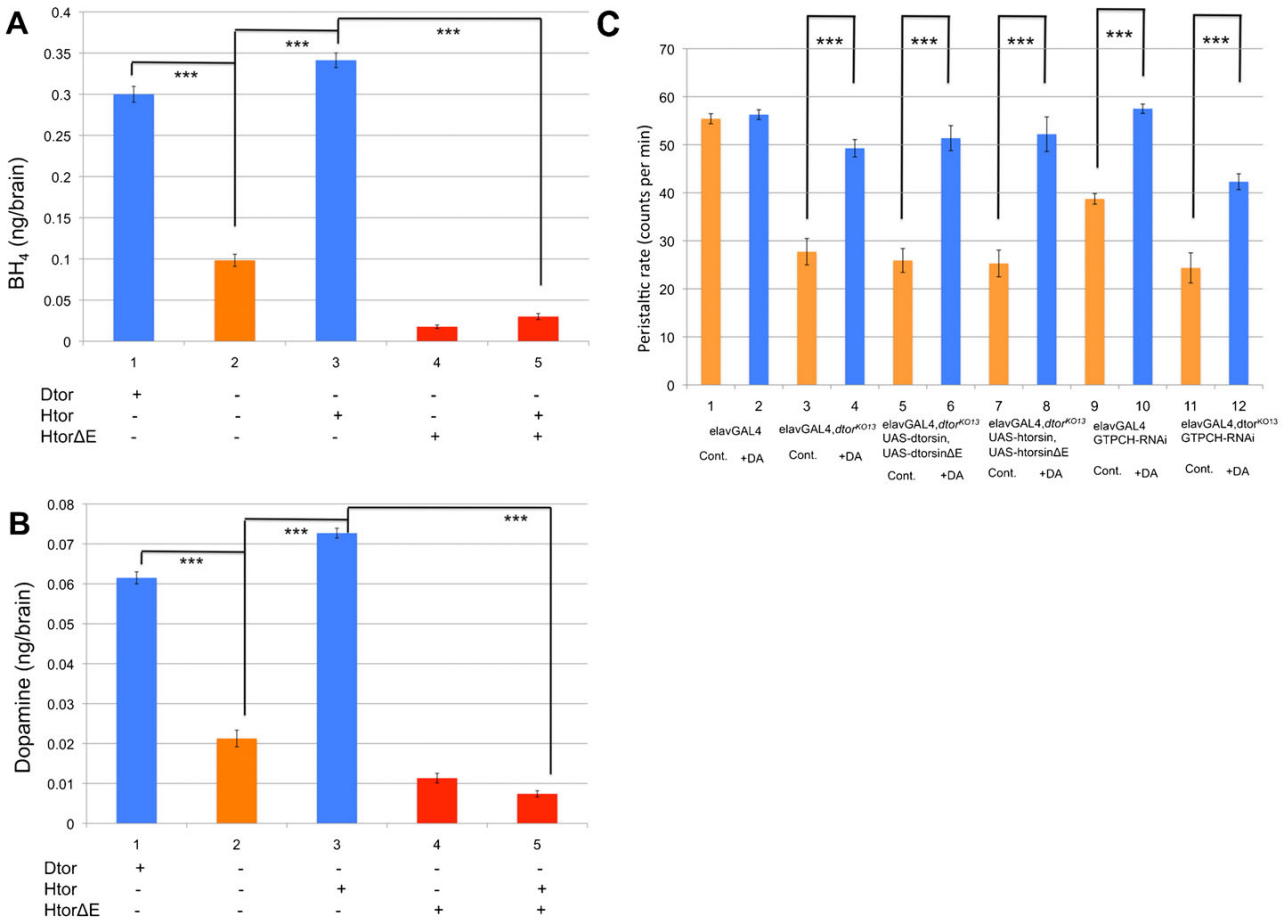
Neuronal expression of human torsinAΔE has a dominant-negative effect on BH_4_ and dopamine levels. (A) Effect of neural expression of human torsinAΔE on BH_4_ pools in larval brains (nanograms per brain). BH_4_ was extracted from third instar brains and separated and quantified by HPLC. The genotypes of larvae were: (1) *y w*/Y males, (2) *w elavGAL4 dtorsin^KO13^*/Y males, (3) *w elavGAL4 dtorsin^KO13^*/Y; UAS-htorsinA/+ males, (4) *w elavGAL4 dtorsin^KO13^*/Y; UAS-htorsinAΔE/+ males, (5) *w elavGAL4 dtorsin^KO13^*/Y; UAS-htorsinA, UAS-htorsinAΔE/+ males. (B) Effect of neural expression of human torsinAΔE on dopamine pools in larval brains (nanograms per brain). Monoamines were extracted from third instar brains and dopamine was separated and quantified by HPLC. The genotypes of larvae were: (1) *y w*/Y (wild type) males, (2) *w elavGAL4 dtorsin^KO13^*/Y (*dtorsin*-null) males, (3) *w elavGAL4 dtorsin^KO13^*/Y; UAS-htorsinA/+ males, (4) *w elavGAL4 dtorsin^KO13^*/Y; UAS-htorsinAΔE/+ males, (5) *w elavGAL4 dtorsin^KO13^*/Y; UAS-htorsinA, UAS-htorsinAΔE/+ males. Results are expressed as the mean±S.E.M. ***p<0.0001. (C) Peristaltic frequencies were counted for the wandering stage third instar larvae. The genotypes are: (1) *w elavGAL4*/Y males without dopamine supplementation (n = 12), (2) *w elavGAL4*/Y males with 20 mM dopamine supplementation (n = 11), (3) *w elavGAL4 dtorsin^KO13^/*/Y males without dopamine supplementation (n = 11), (4) *w elavGAL4 dtorsin^KO13^*/Y males with 20 mM dopamine supplementation (n = 11), (5) *w elavGAL4 dtorsin^KO13^*/Y; UAS*-dtorsinΔE*(#12)(II)/+; UAS-*dtorsin*(A11)(III)/+ males without dopamine supplementation (n = 11), (6) *w elavGAL4 dtorsin^KO13^*/Y; UAS-*dtorsinΔE*(#12)(II)/+; UAS-*dtorsin*(A11)(III)/+ males with 20 mM dopamine supplementation (n = 8), (7) *w elavGAL4 dtorsin^KO13^*/Y; UAS-htorsinA, UAS-htorsinAΔE/+ males without dopamine supplementation (n = 14), (8) *w elavGAL4 dtorsin^KO13^*/Y; UAS-htorsinA, UAS-htorsinAΔE/+ males with 20 mM dopamine supplementation (n = 5), (9) *w elavGAL4*/Y; GTPCH (*Pu*)-RNAi (v107296) males without dopamine supplementation (n = 11), (10) *w elavGAL4*/Y; GTPCH (*Pu*)-RNAi (v107296) males with 20 mM dopamine supplementation (n = 10), (11) *w elavGAL4 dtorsin^KO13^/*/Y; GTPCH (*Pu*)-RNAi (v107296) males without dopamine supplementation (n = 11), (12) *w elavGAL4 dtorsin^KO13^*/Y; GTPCH (*Pu*)-RNAi (v107296) males with 20 mM dopamine supplementation (n = 10). Results are mean±S.E.M. ***p<0.0001, very significant difference between without and with 20 mM dopamine supplementation.

Tyrosine hydroxylase is the rate limiting enzyme in dopamine synthesis (Friggi-Grelin et al., 2003) and its activity is limited by the availability of the BH_4_ cofactor (Kumer and Vrana, 1996). In flies and mammals, activity of GTPCH, the first enzyme in the BH_4_ biosynthesis pathway, controls the intracellular concentration of the cofactor (Kumer and Vrana, 1996; Krishnakumar et al., 2000; Thöny et al., 2000). Thus, dopamine pools are subject to regulation by protein levels and catalytic activity of GTPCH. We have previously reported that there is a significant reduction of GTPCH activity and dopamine levels in larval and adult head of heterozygous *dtorsin^KO13^/+* and *dtorsin^KO78^/+* females (Wakabayashi-Ito et al., 2011). To investigate whether expression of human torsinAΔE could also reduce the dopamine pool level, we measured BH_4_ levels and dopamine levels in extracts from brains of wild type male larvae expressing wild type human torsinA, *dtorsin^KO13^* male larvae expressing human torsinAΔE, and *dtorsin^KO13^* male larvae expressing both human torsinA and human torsinAΔE (Fig. 4A,B). The level of BH_4_ in *dtorsin^KO13^* male brains was significantly lower (0.098±0.007 ng/brain, n = 3 replicate samples, each sample = 75 brains, p<0.001) (Fig. 4A, column 2) compared to wild type brains (0.300±0.010, n = 3 replications) (Fig. 4A, column 1). The BH_4_ level in *dtorsin^KO13^* male brains expressing wild type human torsinA (0.341±0.009, n = 3, p<0.001) (Fig. 4A, column 3) was significantly higher compared to those in *dtorsin^KO13^* male brains (Fig. 4A, column 2). Neuronal expression of human torsinAΔE further decreased BH_4_ levels (0.018±0.002, n = 3, p<0.01) (Fig. 4A, column 4 compared to column 2). Co-expression of human torsinAΔE with wild type human torsinA blocked the rescue by human torsinA (0.030±0.004, n = 3, p<0.001) (Fig. 4A, column 5 compared to column 3).

Similarly, the level of dopamine in *dtorsin^KO13^* male larval brains was significantly lower (0.021±0.002 ng/brain, n = 3, p<0.001) (Fig. 4B, column 2) as compared to wild type brains (0.062±0.002, n = 3) (Fig. 4B, column 1). The dopamine level in *dtorsin^KO13^* male brains expressing wild type human torsinA (0.073±0.001, n = 3, p<0.001) (Fig. 4B, column 3) was significantly higher compared to that in *dtorsin^KO13^* male brains (Fig. 4B, column 2). Neuronal expression of human torsinAΔE in *dtorsin^KO13^* males further decreased dopamine levels (0.011±0.001, n = 3, p<0.01) (Fig. 4B, column 4 compared to column 2). Co-expression of human torsinAΔE with wild type human torsinA dominantly blocked the rescue of dopamine levels by wild type torsinA (0.0074±0.0008, n = 3, p<0.001) (Fig. 4B, column 5 compared to column 3).

### DtorsinΔE dominantly inhibits larval locomotion

**Fig. 5.**
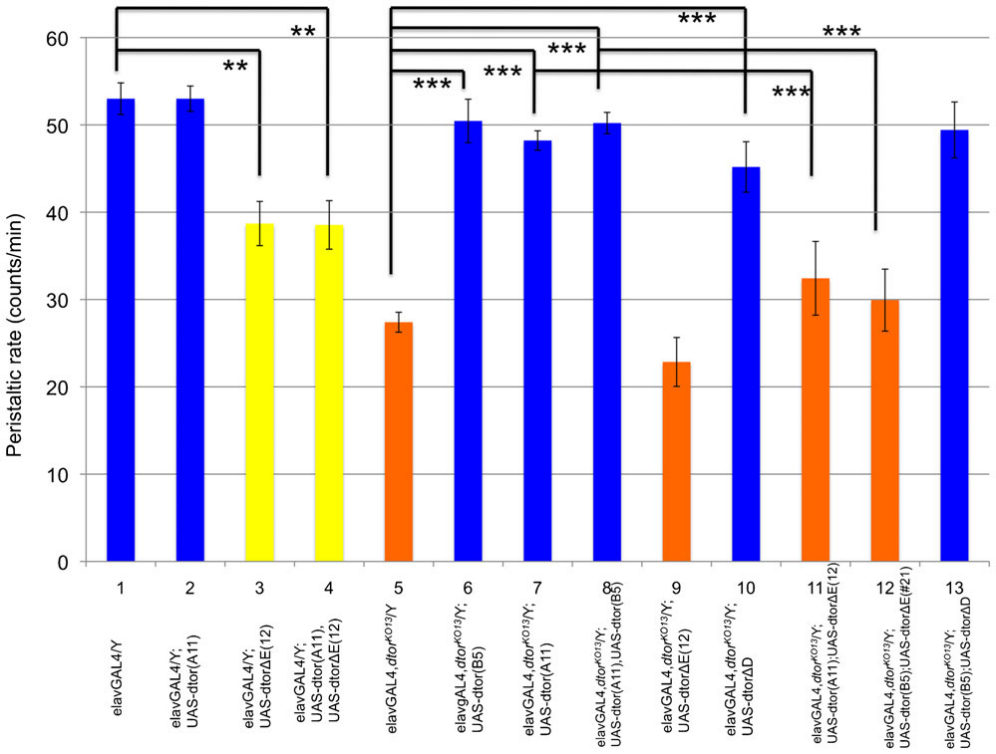
Neuronal expression of *Drosophila* DtorsinΔE has a dominant-negative effect on larval locomotion. Peristaltic frequencies were counted for the wandering stage third instar larvae of the genotype: (1) *w elavGAL4*/Y (wild type) male (n = 9), (2) *w elavGAL4*/Y; UAS-*dtorsin*(A11)(III)/+ male (n = 8), (3) *w elavGAL4*/Y; UAS-*dtorsinΔE*(#12)(II)/+ male (n = 23), (4) *w elavGAL4*/Y; UAS-*dtorsinΔE*(#12)(II)/+; UAS-*dtorsin*(A11)(III)/+ male (n = 15), (5) *w elavGAL4 dtorsin^KO13^*/Y (*dtorsin*-null) male (n = 48), (6) *w elavGAL4 dtorsin^KO13^*/Y; UAS-*dtorsin*(B5)(II)/+ male (n = 20), (7) *w elavGAL4 dtorsin^KO13^*/Y; UAS-*dtorsin*(A11)(III)/+ male (n = 9), (8) *w elavGAL4 dtorsin^KO13^*/Y; UAS-*dtorsin*(B5)(II)/+; UAS-*dtorsin*(A11)(III)/+ male (n = 23), (9) *w elavGAL4 dtorsin^KO13^*/Y; UAS-*dtorsinΔE*(#12)(II)/+ male (n = 13), (10) *w elavGAL4 dtorsin^KO13^*/Y; UAS-*dtorsinΔD*/+ male (n = 11), (11) *w elavGAL4 dtorsin^KO13^*/Y; UAS-*dtorsin*(A11)(III)/+; UAS-*dtorsinΔE*(#12)(II)/+ male (n = 7), (12) *w elavGAL4 dtorsin^KO13^*/Y; UAS-*dtorsin*(B5)(II)/+; UAS-*dtorsinΔE*(#21)(III)/+ male (n = 14), (13) *w elavGAL4 dtorsin^KO13^*/Y; UAS-*dtorsin*(B5)(II)/+; UAS-*dtorsinΔD*/+ male (n = 20). Results are expressed as the means±S.E.M. ***p<0.0001, **p<0.001.

Dtorsin protein has conserved amino acids E306/D307, compared to E302/E303 in human torsinA (supplementary material Fig. S1). To determine whether Dtorsin with either ΔE306 or ΔD307 deleted would have a similar dominant-negative activity on the wild type Dtorsin protein as observed for the human torsinAΔE302/303 mutation, we made two deletion mutant constructs of the *dtorsin* cDNA in E306 (UAS-*dtorsinΔE*) and D307 (UAS-*dtorsinΔD*) and expressed them with the elavGAL4 driver. Although pan-neuronal expression of wild type Dtorsin did not affect larval locomotion in wild type *Drosophila* (peristaltic frequency 53.0±1.5, n = 8, not significant) (Fig. 5, column 2) compared to wild type (53.0±1.8, n = 9) (Fig. 5, column 1), wild type male larvae expressing DtorsinΔE exhibited a significant locomotion deficit (38.7±2.5, n = 23, p = 0.002) (Fig. 5, column 3). Male larvae co-expressing DtorsinΔE and wild type Dtorsin also exhibited a locomotion deficit (38.54±2.8, n = 15, p = 0.0012) (Fig. 5, column 4 compared to column 1) similar to the deficit caused by expression of DtorsinΔE only (column 3).

Mutant male larvae (*dtorsin^KO13^*) expressing wild type Dtorsin showed much improved larval locomotion. We tested two independent transgenic lines expressing wild type Dtorsin. Expression of a second chromosome transgene, UAS-*dtorsin*(B5) in male *dtorsin^KO13^* larvae resulted in a peristaltic frequency of 50.5±2.5, n = 20, p<0.0001 (Fig. 5, column 6), while expression of another transgene UAS-*dtorsin*(A11), on the third chromosome, rescued the peristaltic frequency to 48.2±1.1, n = 9, p<0.0001 (Fig. 5, column 7) compared to the *dtorsin*-null (*dtorsin^KO13^*) males (Fig. 5, column 5). The presence of two copies of UAS-*dtorsin* transgenes (B5 and A11) together in the *dtorsin*-null background did not elevate locomotion further (peristaltic frequency: 50.2±1.2, n = 23, p<0.0001) (Fig. 5, column 8).

In striking contrast to the rescuing effect of wild type Dtorsin expression, DtorsinΔE expression in male *dtorsin^KO13^* larvae failed to rescue the locomotion deficit (22.9±2.8, n = 13, not significant) with a slight reduction of peristaltic rate (Fig. 5, column 9), relative to *dtorsin^KO13^* males (Fig. 5, column 5). Similarly, mutant males co-expressing DtorsinΔE and wild type Dtorsin transgenes exhibited a locomotion deficit that was not significantly different from that of the *dtorsin^KO13^* larvae (UAS-*dtorsin*(A11) and UAS-*dtorsin*ΔE(#12); 32.4±4.2, n = 7, not significant) (Fig. 5, column 11); UAS-*dtorsin*(B5) and UAS-*dtorsin*ΔE(#21): 29.9±3.6, n = 14, not significant) (Fig. 5, column 12).

Interestingly, pan-neuronal expression of DtorsinΔD in *dtorsin^KO13^* males rescued the larval mobility (45.2±2.9, n = 11, p<0.0001) (Fig. 5, column 10 compared to column 5). Similarly, co-expression of DtorsinΔD with the wild type Dtorsin in *dtorsin^KO13^* males had no effect on locomotion (peristaltic frequency: 49.4±3.2, n = 20) (Fig. 5, column 13). These results indicate that E302/303 of human torsinA protein and E306 of *Drosophila* Dtorsin protein are functionally similar and that deletion of these glutamates both cause reduced locomotion in *Drosophila* larvae, presumably due to the same functional abnormality, while DtorsinΔD appears similar to wild type Dtorsin.

### DtorsinΔE dominantly suppresses GTPCH expression

**Fig. 6.**
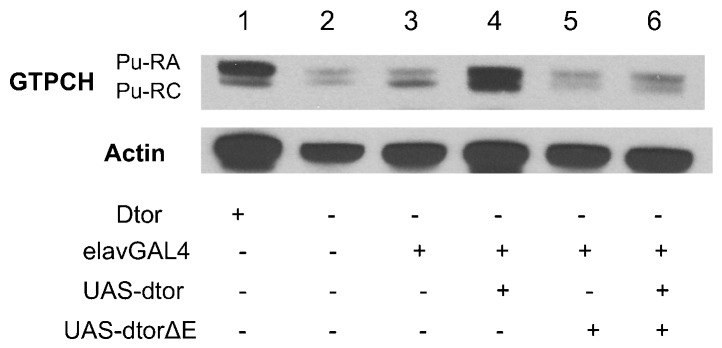
Neuronal expression of *Drosophila* DtorsinΔE has a dominant-negative effect on GTPCH protein levels. Adult head extracts were analyzed by western blots. The membrane was probed with rabbit anti-GTPCH A/C (upper panel) and reprobed with rabbit anti-actin (lower panel). The genotypes are: (1) *y w*/Y (wild type) males, (2) *y w dtorsin^KO13^*/Y (*dtorsin*-null) males, (3) *w elavGAL4 dtorsin^KO13^*/Y (*dtorsin*-null) males, (4) *w elavGAL4 dtorsin^KO13^*/Y; UAS-*dtorsin*(B5)(II)/+ males, (5) *w elavGAL4 dtorsin^KO13^*/Y; UAS- *dtorsinΔE*(#12)(III)/+ males, (6) *w elavGAL4 dtorsin^KO13^*/Y; UAS-*dtorsin*(B5)(II): UAS-*dtorsinΔE*(#12)(III)/+ males. The locations of GTPCH (Pu-RA: 45 kDa, Pu-RC: 43 kDa) and actin (42 kDa) are indicated. Thirty µg of proteins were loaded in each lane.

These studies described above demonstrate a striking similarity in the dominant inhibition of larval locomotion by DtorsinΔE and human torsinAΔE. Since we found that human torsinAΔE dominantly inhibited GTPCH protein expression, we next examined the protein levels of GTPCH in adult male heads expressing wild type Dtorsin and DtorsinΔE in the *dtorsin*-null background (Fig. 6). The expression of endogenous GTPCH in the *dtorsin^KO13^* mutant line and in the *dtorsin^KO13^* elavGAL4 transgene line revealed similar patterns of reduced GTPCH expression of both RA and RC isoforms (Fig. 6, lane 2 and 3). Dtorsin expressed in *dtorsin^KO13^* males, under the control of elavGAL4, rescued GTPCH expression substantially (Fig. 6, lane 4, compared to lane 3; supplementary material Fig. S6 column 4, compared to column 3).

DtorsinΔE expressed in *dtorsin^KO13^* neurons failed to affect the GTPCH protein level (Fig. 6, lane 5, compared to lane 3; supplementary material Fig. S6, column 5, compared to column 3). Severe reduction of GTPCH was also observed in adult males co-expressing Dtorsin and DtorsinΔE in *dtorsin^KO13^* (Fig. 6, lane 6, compared to lane 4; supplementary material Fig. S6, column 6, compared to column 4). These results demonstrate that DtorsinΔE and human torsinAΔE have indistinguishable effects on the expression of GTPCH protein in *Drosophila* brains, both dominantly inhibiting GTPCH expression. In contrast, DtorsinΔD expressed in *dtorsin^KO13^* neurons moderately rescued GTPCH protein level (supplementary material Fig. S7), consistent with the results of larval locomotion assays (Fig. 5).

### The mobility defect of larvae expressing either human torsinAΔE or DtorsinΔE can be rescued by dopamine supplementation

**Fig. 7.**
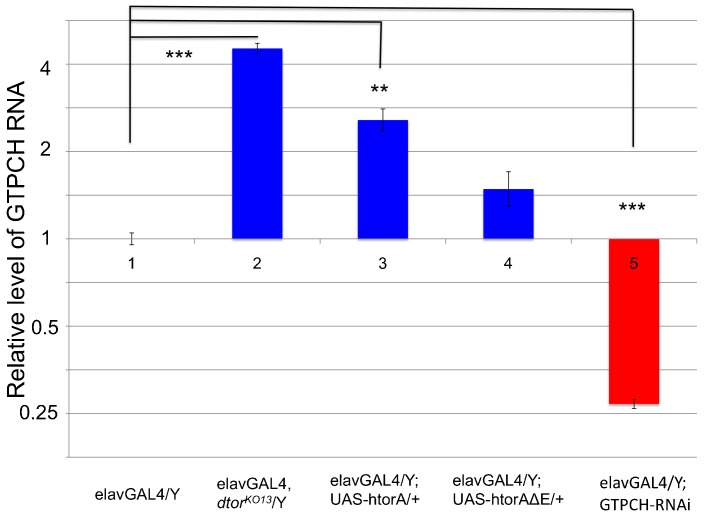
*Drosophila dtorsin*-null mutation does not reduce GTPCH mRNA levels. Total RNA was extracted from adult heads. Relative amount of GTPCH RNA was calculated compared to the internal control, *RpL32* (*rp49*) RNA in each sample. The genotypes are: (1) *w elavGAL4*/Y males (n = 3), (2) *w elavGAL4 dtorsin^KO13^*/Y (*dtorsin*-null) males (n = 3), (3) *w elavGAL4*/Y; UAS-htorA/+ males (n = 3), (4) *w elavGAL4*/Y; UAS-htorAΔE/+ males (n = 3), and (5) w elavGAL4/Y; GTPCH (*Pu*)-RNAi (v107296) males (n = 3). Results are expressed as the means±S.E.M. ***p<0.0001, **p<0.001.

In *Drosophila*, ingestion of dopamine increases dopamine pools in the fly head, though in mammals peripheral dopamine does not enter the brain (Chaudhuri et al., 2007). We previously showed that the locomotor deficit phenotype in *dtorsin^KO13^* mutant male was partially rescued by dopamine supplementation to the larval growth medium, but not by serotonin or octopamine (Wakabayashi-Ito et al., 2011). Since we observed in the current study a very similar reduction of dopamine levels in larval brains expressing human torsinAΔE, we hypothesized that dopamine supplementation to the larval growth medium could also restore the locomotion defect of larvae expressing human torsinAΔE (or *Drosophila* DtorsinΔE). To test this hypothesis, we added 20 mM dopamine in the food of larvae with different *dtorsin* genotypes (Fig. 4C). Dopamine supplementation had no effect on the locomotion of wild type (elavGAL4/Y) larvae (56.3±1.0, n = 11, p = 0.562) (Fig. 4C, column 2) compared to the larvae of the wild type without dopamine (55.4±1.0, n = 12) (Fig. 4C, column 1). In contrast, dopamine supplementation substantially rescued the locomotion defect of *dtorsin^KO13^* larvae (49.3±1.8, n = 11, p<0.0001) (Fig. 4C, column 4) compared to the larvae of the same genotype without dopamine (27.7±2.8, n = 11) (Fig. 4C, column 3) confirming our previous results (Wakabayashi-Ito et al., 2011). Dopamine supplementation also rescued the locomotion defect of *dtorsin^KO13^* larvae expressing DtorsinΔE and wild type Dtorsin (51.4±2.6, n = 8, p<0.0001) (Fig. 4C, column 6) compared to the larvae of the same genotype without dopamine (25.9±2.5, n = 11) (Fig. 4C, column 5). Similarly, dopamine supplementation also substantially rescued the locomotion defect of *dtorsin^KO13^* larvae expressing human torsinAΔE and wild type torsinA (52.2±3.6, n = 5, p<0.0001) (Fig. 4C, column 8) compared to the larvae of the same genotype without dopamine (25.3±2.8, n = 14) (Fig. 4C, column 7). These results demonstrate that locomotor defects caused by the pan-neuronal expression of human torsinAΔE or DtorsinΔE, measured by our larval locomotion assay, can be substantially rescued by dopamine supplementation. As a control, knockdown of GTPCH (*Pu*) mRNA expression levels was accomplished by neuronal expression of GTPCH RNAi, a short-hairpin specific for GTPCH (*Pu*) gene (Fig. 7), which was accompanied by a moderate reduction of larval locomotion (38.7±1.1, n = 11) (Fig. 4C, column 9). Dopamine supplementation almost completely rescued locomotion defect of wild type larvae expressing GTPCH RNAi (57.5±1.0, n = 10, p<0.0001) (Fig. 4C, column 10). Dopamine supplementation also substantially rescued the locomotion defect of *dtorsin^KO13^* larvae expressing GTPCH RNAi (42.3±1.7, n = 10, p<0.0001) (Fig. 4C, column 12) compared to the larvae of the same genotype without dopamine (24.4±3.1, n = 11) (Fig. 4C, column 11).

### The expression level of GTPCH is regulated at the post-transcriptional level

Having found that Dtorsin/torsinA regulates GTPCH protein, we next tested whether torsin regulates GTPCH expression at the transcriptional or post-transcriptional level. In order to analyze these alternative possibilities, we prepared total RNA from adult brains and analyzed GTPCH (*Pu*) mRNA levels by quantitative RT-PCR (qRT-PCR). The relative amount of GTPCH mRNA was determined by normalizing to mRNA for the housekeeping gene *RpL32* (*rp49*) as an internal control (see Materials and Methods). A significant increase, rather than reduction of GTPCH mRNA levels was observed in the brains of *elavGAL4 dtorsin^KO13^*/Y (4.52±0.20, n = 3, p<0.0001) (Fig. 7, column 2) compared to those in wild type: elavGAL4/Y (1.00, n = 3) (Fig. 7, column 1). Expression of wild type human torsinA (2.57±0.24, n = 3, p = 0.0007) (Fig. 7, column 3) substantially increased GTPCH mRNA levels, while mutant human torsinAΔE (1.48±0.23, n = 3, p = 0.534, not significant) (Fig. 7, column 4) had no effect on GTPCH mRNA levels. Neuronal expression of GTPCH RNAi substantially reduced GTPCH RNA levels (0.27±0.01, n = 3, p<0.0001) (Fig. 7, column 5), validating our quantification of GTPCH mRNA by qRT-PCR. There were some variations in the relative abundance of GTPCH mRNA, but the reason is not clear at this moment. These results, however, indicate that *dtorsin/torsin* mutant brains do not have decreased levels of GTPCH mRNA and therefore have a defect in GTPCH expression at the post-transcriptional level.

## Discussion

*Drosophila* has a single torsin-related gene, *dtorsin* (*Torsin*), with 31.9% amino acid identity to human torsinA (supplementary material Fig. S1). *dtorsin*-null animals have reduced locomotion at the third instar larval stage and reduced pigmentation in the adult stage (Wakabayashi-Ito et al., 2011). The dopamine levels and GTPCH activity/protein levels are severely reduced in *dtorsin*-null animals, suggesting GTPCH deficiency is responsible for dopamine depletion since TH protein is unaffected in the mutant (Wakabayashi-Ito et al., 2011). The pan-neuronal expression of wild type *Drosophila* Dtorsin or human torsinA rescued the locomotion defect in *dtorsin*-null larvae and adults, suggesting that human torsinA and *Drosophila* Dtorsin are functionally conserved (Figs 1, 5) (Wakabayashi-Ito et al., 2011). Pan-neuronal expression of human torsinAΔE protein alone did not rescue the locomotion defect, or the depletion of GTPCH protein, BH_4_, and dopamine (Figs 1–4) in *dtorsin*-null larvae and adults, demonstrating that human torsinAΔE protein is inactive. Further, co-expression of human wild type torsinA and torsinAΔE did not rescue the defects (Figs 1–4), demonstrating a dominant-negative effect of torsinAΔE on wild type torsinA activity. These results, for the first time, clearly show that torsinAΔE inhibits wild type torsinA activity in neurons, resulting in reduced locomotion and dopamine levels in *Drosophila*.

*Drosophila* Dtorsin has similar types of amino acids E306-D307 compared to human torsinA E302-E303 in the conserved location near the C terminal region of the protein (supplementary material Fig. S1). DtorsinΔE306, when expressed in neurons, had a similar dominant-negative effect on locomotion and GTPCH protein levels as human torsinAΔE (Figs 5, 6), while DtorsinΔD307 was still active as it could rescue the *dtorsin*-null locomotion defect as well as GTPCH protein expression, and had no inhibitory effect on wild type Dtorsin (Fig. 5; supplementary material Fig. S7). Furthermore, neuronal expression of DtorsinΔE306 inhibited locomotion of wild type larvae, demonstrating a dominant-negative effect on the wild type protein. The phenotypes caused by neuronal expression of human torsinAΔE or *Drosophila* DtorsinΔE are indistinguishable from those of *dtorsin*-null larvae or adults, resulting in co-reduction of locomotion, dopamine levels, and GTPCH protein levels. These results strongly support the hypothesis that torsinAΔE acts as a dominant-negative molecule that suppresses the wild type protein activity (Breakefield et al., 2001).

We have demonstrated that *dtorsin*-null larvae and DtorsinΔE (or human torsinAΔE) expressing larvae have very similar phenotypes, resulting in the severely decreased level of GTPCH. Rates of dopamine synthesis depend on the activity of TH, which in turn depends on the amount of BH_4_ produced by GTPCH (O'Donnell et al., 1989; Thöny et al., 2000). Severe reduction of GTPCH protein levels results in a shortage of BH_4_ and decreased activity of TH, thereby leading to decreased dopamine pool levels in brains of *dtorsin*-null animals (Wakabayashi-Ito et al., 2011), as well as in brains expressing torsinAΔE (Fig. 4A,B). This defect, however, is unlikely to be the only defect in the dopamine signal transduction system in *dtorsin*-null or torsinAΔE-expressing animals. Although feeding dopamine could partially rescue the locomotion defect in our assay in *dtorsin*-null larvae (Wakabayashi-Ito et al., 2011) or in DtorsinΔE (or human torsinAΔE)-expressing larvae (Fig. 4C), very few larvae of *dtorsin*-null or torsinAΔE-expressing animals survived until the late third instar larval stage (data not shown). Early lethality could be the result of earlier developmental requirements for dopamine since strong loss-of-function mutations in the TH-encoding gene cause embryonic lethality in *Drosophila* (Neckameyer and White, 1993). Alternatively, Dtorsin may be affecting other neurotransmitter signaling systems directly or indirectly through dysfunction in dopaminergic circuitry. In the case of DYT1 dystonia patients, L-dopa is not therapeutic, suggesting that dopamine cannot compensate for defects resulting from mutant torsinA (Breakefield et al., 2008). Recent publications in mouse DYT1 model systems demonstrated defective dopamine D2 receptor signaling in the striatal cholinergic neurons (Sciamanna et al., 2009; Sciamanna et al., 2011; Sciamanna et al., 2012). The lack of responsiveness of DYT1 patients to L-dopa treatment would be expected if the dopamine D2 receptor signaling or other component of the dopaminergic system is defective in addition to defects in dopamine synthesis.

Translational control of localized mRNA is a common mechanism for regulating protein expression in specific subdomains of a cell, in processes such as body axis formation, asymmetric cell division and synaptic plasticity (St Johnston, 2005; Holt and Bullock, 2009; Medioni et al., 2012). These localized mRNAs are often transported in large ribonucleoprotein particles (RNPs) or RNA granules (Kiebler and DesGroseillers, 2000; Kiebler and Bassell, 2006; Holt and Bullock, 2009; Medioni et al., 2012). We have recently shown that *dtorsin* is involved in export of large RNPs out of nuclei on the way to the neuromuscular junction (Jokhi et al., 2013). Here, we have shown that the mRNA levels of GTPCH/*Punch* gene were not significantly decreased in *dtorsin*-null adult brains (Fig. 7), suggesting that the regulation of GTPCH/*Punch* expression is at the post-transcriptional level. This is consistent with a model in which GTPCH mRNA is transported through the nuclear membrane as a part of a large RNP complex whose transport depends on Dtorsin. If this hypothesis is correct, the Dtorsin protein could regulate the nuclear export and subsequent transport of large RNP complexes with subsequent compromise of the translation of the GTPCH mRNA. This nuclear export of mRNAs within RNPs could explain the mechanism by which torsin regulates expression of multiple proteins such as GTPCH and dopamine D2 receptor at the same time and thereby modulate synaptic plasticity (Sciamanna et al., 2012). Further testing of this hypothesis will be very important for understanding the molecular function of torsin proteins and the pathophysiology of DYT1 disease in human patients. The *Drosophila* system with its abundant genetic tools provides us an excellent model system to probe this hypothesis.

## Materials and Methods

### Fly stocks

Flies were grown on standard medium containing cornmeal, yeast and agar at 25°C in fly incubators with a constant humidity of 70% (Ashburner and Roote, 2007). ElavGAL4 transgenic strain was obtained from the Drosophila Stock Center (Bloomington, IN USA). The *dtorsin*-null lines, *y w dtorsin^KO13^*/FM7i, Act-GFP and *w elavGAL4 dtorsin^KO13^*/FM7i, Act-GFP were described previously (Wakabayashi-Ito et al., 2011). The *Punch*-null line, *Pu^Z22^*, was previously described (Mackay et al., 1985). The RNAi line for GTPCH (*Pu*) gene, v107296 (KK107763) (Dietzl et al., 2007), was obtained from Vienna Drosophila RNAi Stock Center (Vienna, Austria). This RNAi line has 514 nt hairpin sequences that target all three isoforms (Pu-RA, Pu-RB, and Pu-RC) of GTPCH (*Pu*) transcripts.

### UAS lines

*dtorsin*ΔE and *dtorsin*ΔD cDNA constructs were made from the wild type *dtorsin* cDNA using QuikChange II XL Site-Directed Mutagenesis kit (Agilent Technologies, Santa Clara, CA USA). Briefly, a 1.2 kb wild type *dtorsin* cDNA was cut from pUAST-*dtorsin* with EcoRI and NotI (Wakabayashi-Ito et al., 2011) and cloned between the EcoRI and NotI sites of pBluescript II KS (Agilent Technologies). Mutagenesis strand synthesis was done following the manufacturer's protocol using two primers torp4aE3 (5′-CTAATGGAGGAGTTTATTATGTCAATGATTTTTTGGTTGTTCGC-3′) and torp4aE5 (5′-GCGAACAACCAAAAAATCATTGACATAATAAACTCCTCCATTAG-3′) to make *dtorsin* cDNA that lacks GAG (E306), and torp4aD3 (5′-CTAATGGAGGAGTTTATTATCTCAATGATTTTTTGGTTGTTCGC-3′) and torp4aD5 (5′-GCGAACAACCAAAAAATCATTGAGATAATAAACTCCTCCATTAG-3′) to make *dtorsin* cDNA that lacks GAC (D307), respectively. After confirming mutated sequences, the insert was again cut out with *EcoRI* and *NotI* and inserted between *EcoRI* and *NotI* sites of pUAST to produce pUAST-*dtorsinΔE* and pUAST-*dtorsinΔD*. The transgenic lines E12 (pUAST-*dtorsinΔE* transgene on the second chromosome), E21 (pUAST-*dtorsinΔE* on the third chromosome) and D19 (pUAST-*dtorsinΔD* on the third chromosome) were used for the experiments.

A 1.0 kb human torsinAΔE cDNA was amplified from pcDNA3-htorM (Hewett et al., 2000) by PCR using the following primers htor5 (5′-GCGGGATCCATTCATGAAGCTGGGCCGGGCCGTGCTGGGCCTGC-3′) and htor3 (5′-CTCGAGCGGCCGCTCAATCATCGTAGTAATAATCTAACTTGGTG-3′). The PCR product was digested with *Acc65I* and *NotI* and inserted between *Acc65I* and *NotI* sites of pUAST. Injections were performed by Genetic Services, Inc. (Cambridge, MA USA). The transgenic line #24 with UAS-htorsinAΔE transgene on the second chromosome was used for the experiments.

### Larval locomotion assay

The larval locomotion assay was done as described previously (Wakabayashi-Ito et al., 2011). Briefly, a wandering third instar larva of a particular genotype was individually picked from the vial with a bamboo stick and placed at the center of a 100 mm petri dish containing 0.7% agarose at room temperature placed on a light box. Larval locomotion was recorded for one minute using a Canon Powershot G7 digital camera attached to a stereoscopic microscope. Peristaltic frequency was counted manually using the Quicktime movie. The experiments were done in a double-blinded manner with only numbers assigned for each genotype. Peristaltic rates are usually highly reproducible with little variation for each genotype with relatively small SEM values. Since we were unable to get *dtorsin*-null homozygous females (Wakabayashi-Ito et al., 2011), we used *dtorsin*-null males for locomotion assays. We did not observe any significant gender difference between males and females of wild type larvae in our locomotion assay.

### Adult locomotion assay

The adult locomotion assay was adapted from the method described previously (Carbone et al., 2006). Flies were maintained at 25°C and a 12 hour light–12 hour dark circadian cycle. At least 10 males of each genotype, aged 3–5 days post-eclosion, were assayed in a double-blind manner. Individual flies were placed in vials and allowed to acclimate to the vial for 1 hour prior to assay. The vials were subjected to a gentle mechanical disturbance, and then locomotion behavior was quantified as the number of seconds each fly spent in motion during a 45 second period. The experiments were done in a double-blinded manner with only numbers assigned for each genotype. Each assay was replicated five times per fly. All assays were completed at the same time of the day (12 pm–3 pm). The results are usually highly reproducible with little variation with relatively small SEM values.

### HPLC analysis

Dopamine and BH_4_ were separated by HPLC using a CoulArray HPLC system (model 5600A; ESA, Chelmsford, MA USA) and a Synergi 4 µm Hydro-RP column (4.6×150 mm; Phenomenex, Torrance, CA), as described (Chaudhuri et al., 2007). Brains of third instar larvae or heads of 48–72 hour post-eclosion adult flies were homogenized in 0.1 M perchloric acid. One hundred third instar larval brains or 75 to 200 adult heads were extracted in 100–200 µl of 0.1 M perchloric acid. Ten microliters of each extract were injected for each sample. Pool sizes were determined relative to freshly prepared standards (Sigma-Aldrich, St. Louis, MO USA). Analysis was performed using ESA CoulArray software.

### Western blot analysis

Detection of proteins in adult heads (Figs 2, 6; supplementary material Figs S3, S6, S7) was performed, as described (Wakabayashi-Ito et al., 2011). Briefly, fifty heads from adult males of each genotype were homogenized in 100 µl RIPA buffer [50 mM Tris-HCl, pH 8.0, 150 mM NaCl, 1% NP40, 0.5% deoxycholate, 0.1% SDS (sodium dodecyl sulfate)] with Protein Inhibitor Cocktail (Roche Applied Science, Indianapolis, IN USA). The proteins (30 µg), which corresponded to approximately three adult heads, were separated by electrophoresis in 10% SDS-polyacrylamide gels and transferred to Protran BA85 (0.45 µm pore size) nitrocellulose membranes (Sigma-Aldrich). Membranes were blocked with 10% non-fat dry milk in TBST (20 mM Tris-HCl buffer, pH 7.6, 167 mM sodium chloride, 0.1% Tween 20) and incubated with antibodies in 5% non-fat dry milk in TBST. GTPCH protein was detected using affinity-purified polyclonal anti-GTPCH isoform A/C antibody (Chen et al., 1994) at 1:50,000 dilution. Human torsinA protein was detected using rabbit polyclonal anti-human torsinA TA-2 (Bragg et al., 2004) at 1:5000 dilution, Rabbit anti-actin antibody (Sigma-Aldrich) was used at 1:5000 dilution. The secondary antibody used was peroxidase-conjugated anti-rabbit IgG at 1:5000 dilution (Jackson ImmunoResearch, West Grove, PA USA). Signals were detected using Supersignal West Pico Chemiluminescent Substrate (Thermo Fisher Scientific, Waltham, MA USA).

For detection of proteins in larval brains (Fig. 3; supplementary material Figs S4, S5), fifteen whole male brains from each genotype were dissected from late third instar larvae in phosphate-buffered saline (PBS) and homogenized in 50 µl of RIPA lysis buffer (AMRESCO)/2 mM DTT/1× protease inhibitor cocktail (AMRESCO, Solon, OH USA) containing 2 mM EDTA added immediately before use. Forty µl of supernatant were mixed with 14.9 µl of NuPage LDS sample buffer (Life Technologies, Carlsbad, CA USA) and 5.7 µl of 500 mM DTT. Twenty µg of proteins per lane were separated by electrophoresis on 4–12% NuPage Bis-Tris mini gels (Life Technologies). Separated proteins were transferred into a nitrocellulose membrane, and blocked with 2.5% BSA in TBST. Rabbit anti-GTPCH isoform A/C was used at 1:8000, while mouse anti-syntaxin (8C3 supernatant, Developmental Studies Hybridoma Bank) (1:200) was used in 5% BSA in TBST. Horseradish peroxidase-conjugated anti-rabbit (1:20,000) and anti-mouse (1:20,000) IgG secondary antibodies (VWR International, Randor, CA USA) were prepared in 2.5% BSA in TBST.

After the reaction with the peroxidase substrate, the membranes were exposed with multiple exposure times and the optimal condition for densitometry measurement was determined. Relative densities of GTPCH bands (two major species, RA and RC, were combined) and human torsinA bands were quantified from scanned images of X-ray films by the NIH ImageJ 1.40 g software (“gels” function under “analyze” command; http://rsb.info.nih.gov/ij/docs/guide/146-30.html#sec:Analyze-Menu) using densities of actin bands or syntaxin bands as internal standards.

### Isolation of RNA

Total RNA was extracted from thirty adult male fly heads suspended in 100 µl PBS/0.1% Triton X-100 using 800 µl TRI reagent (Molecular Research Center, Inc., Cincinnati, OH USA) and 80 µl of BCP (Molecular Research Center, Inc.) following the manufacturer's protocol.

### qRT-PCR analysis

qRT-PCR was performed as described previously with minor modifications (Balaj et al., 2011). Total RNA (2 µg) was converted into cDNA with the Omniscript reverse transcription kit (Qiagen, Valencia, CA USA) using random primers, according to manufacturer's recommendations, and a 1:10 fraction (corresponding to 2.5 ng reverse transcribed RNA) was used for qRT-PCR. All reactions were performed in a 20 µl reaction using Power SYBR Green PCR Master Mix (Life Technologies) and 320 nM of each primer. Amplification conditions consisted of: 1 cycle of 50°C, 2 minutes; 1 cycle of 95°C, 10 minutes; 40 cycles of 95°C, 15 seconds; and 60°C, 1 minute followed by a dissociation curve analysis of each amplicon on the 7000 ABI Prism PCR system (Life Technologies). Ct values were analyzed in auto mode. The Ct-values were normalized to the housekeeping gene RpL32 (rp49) in each sample (Brown et al., 2009; Willis et al., 2010). The following primers were used for qRT-PCR: *Rp49*: F:5′-CCCAAGGGTATCGACAACAG-3′; R:5′-GTTCGATCCGTAACCGATGT-3′; *Pu*: F:5′-CGGATAGTGATGGCCACGAG-3′; R:5′-AGTAGACGATACGAGCGTGC-3′.

### Dopamine feeding assay

The dopamine feeding assay was done with some modification, as described previously (Wakabayashi-Ito et al., 2011). Fifty females of *w, elavGAL4, dtorsin^KO13^*/*FM7i*, *Actin-GFP* were mated with twenty-five males of *w*; UAS-*dtorsin*(A11)(II);UAS-*dtorsinΔ*E(12)(III) or *w*; UAS-htorsinA(#8), UAS-htorsinAΔE(#24)(II). Fifty Green Fluorescent Protein (GFP)-negative first instar larvae were transferred to 1.5 g Formula 4–24 Instant Drosophila Medium (Carolina Biological Supply Company, Burlington, SC USA) in 7 ml water or 7 ml of 20 mM dopamine hydrochloride (Sigma-Aldrich) solution. Experiments with other genotypes were performed similarly with the same number of larvae in each vial. Larval locomotion assays were performed as described above (Wakabayashi-Ito et al., 2011).

### Statistical analysis

Since we were comparing two groups with comparable genetic backgrounds with the only exception being that of the particular genotype on which we focused, an unpaired t test was used rather than ANOVA. Means of two groups were compared by an unpaired t test using the statistical software Graphpad Prism 5.0 (GraphPad Software, Dan Diego, CA USA).

## Supplementary Material

Supplementary Material

## References

[b1] Ashburner M. Roote J. (2007). Culture of Drosophila: the laboratory setup. Cold Spring Harb. Protoc. 2007, pdb.ip34 10.1101/pdb.ip34 21357029

[b2] Atai N. A. Ryan S. D. Kothary R. Breakefield X. O. Nery F. C. (2012). Untethering the nuclear envelope and cytoskeleton: biologically distinct dystonias arising from a common cellular dysfunction. Int. J. Cell Biol. 2012, 634214 10.1155/2012/634214 22611399 PMC3352338

[b3] Augood S. J. Keller-McGandy C. E. Siriani A. Hewett J. Ramesh V. Sapp E. DiFiglia M. Breakefield X. O. Standaert D. G. (2003). Distribution and ultrastructural localization of torsinA immunoreactivity in the human brain. Brain Res. 986, 12–21. 10.1016/S0006-8993(03)03164-0 12965225

[b4] Balaj L. Lessard R. Dai L. Cho Y.-J. Pomeroy S. L. Breakefield X. O. Skog J. (2011). Tumour microvesicles contain retrotransposon elements and amplified oncogene sequences. Nat. Commun. 2, 180 10.1038/ncomms1180 21285958 PMC3040683

[b5] Bellen H. J. Tong C. Tsuda H. (2010). 100 years of Drosophila research and its impact on vertebrate neuroscience: a history lesson for the future. Nat. Rev. Neurosci. 11, 514–522. 10.1038/nrn2839 20383202 PMC4022039

[b6] Bragg D. C. Camp S. M. Kaufman C. A. Wilbur J. D. Boston H. Schuback D. E. Hanson P. I. Sena-Esteves M. Breakefield X. O. (2004). Perinuclear biogenesis of mutant torsin-A inclusions in cultured cells infected with tetracycline-regulated herpes simplex virus type 1 amplicon vectors. Neuroscience 125, 651–661. 10.1016/j.neuroscience.2004.01.053 15099679

[b7] Bragg D. C. Armata I. A. Nery F. C. Breakefield X. O. Sharma N. (2011). Molecular pathways in dystonia. Neurobiol. Dis. 42, 136–147. 10.1016/j.nbd.2010.11.015 21134457 PMC3073693

[b8] Breakefield X. O. Kamm C. Hanson P. I. (2001). TorsinA: movement at many levels. Neuron 31, 9–12. 10.1016/S0896-6273(01)00350-6 11498045

[b9] Breakefield X. O. Blood A. J. Li Y. Hallett M. Hanson P. I. Standaert D. G. (2008). The pathophysiological basis of dystonias. Nat. Rev. Neurosci. 9, 222–234. 10.1038/nrn2337 18285800

[b10] Brown A. E. Baumbach J. Cook P. E. Ligoxygakis P. (2009). Short-term starvation of immune deficient Drosophila improves survival to gram-negative bacterial infections. PLoS ONE 4, e4490 10.1371/journal.pone.0004490 19221590 PMC2637427

[b11] Brüggemann N. Klein C. (2010). Genetics of primary torsion dystonia. Curr. Neurol. Neurosci. Rep. 10, 199–206. 10.1007/s11910-010-0107-5 20425035

[b12] Carbone M. A. Jordan K. W. Lyman R. F. Harbison S. T. Leips J. Morgan T. J. DeLuca M. Awadalla P. Mackay T. F. (2006). Phenotypic variation and natural selection at catsup, a pleiotropic quantitative trait gene in Drosophila. Curr. Biol. 16, 912–919. 10.1016/j.cub.2006.03.051 16682353 PMC10766118

[b13] Chaudhuri A. Bowling K. Funderburk C. Lawal H. Inamdar A. Wang Z. O'Donnell J. M. (2007). Interaction of genetic and environmental factors in a Drosophila parkinsonism model. J. Neurosci. 27, 2457–2467. 10.1523/JNEUROSCI.4239-06.2007 17344383 PMC6672491

[b14] Chen X. Reynolds E. R. Ranganayakulu G. O'Donnell J. M. (1994). A maternal product of the Punch locus of Drosophila melanogaster is required for precellular blastoderm nuclear divisions. J. Cell Sci. 107, 3501–3513. 7706401 10.1242/jcs.107.12.3501

[b15] Defazio G. (2010). The epidemiology of primary dystonia: current evidence and perspectives. Eur. J. Neurol. 17, **Suppl. 1** 9–14. 10.1111/j.1468-1331.2010.03053.x 20590802

[b16] Dietzl G. Chen D. Schnorrer F. Su K. C. Barinova Y. Fellner M. Gasser B. Kinsey K. Oppel S. Scheiblauer S. (2007). A genome-wide transgenic RNAi library for conditional gene inactivation in Drosophila. Nature 448, 151–156. 10.1038/nature05954 17625558

[b17] Friggi-Grelin F. Coulom H. Meller M. Gomez D. Hirsh J. Birman S. (2003). Targeted gene expression in Drosophila dopaminergic cells using regulatory sequences from tyrosine hydroxylase. J. Neurobiol. 54, 618–627. 10.1002/neu.10185 12555273

[b18] Goodchild R. E. Kim C. E. Dauer W. T. (2005). Loss of the dystonia-associated protein torsinA selectively disrupts the neuronal nuclear envelope. Neuron 48, 923–932. 10.1016/j.neuron.2005.11.010 16364897

[b19] Hanson P. I. Whiteheart S. W. (2005). AAA+ proteins: have engine, will work. Nat. Rev. Mol. Cell Biol. 6, 519–529. 10.1038/nrm1684 16072036

[b20] Hewett J. Gonzalez-Agosti C. Slater D. Ziefer P. Li S. Bergeron D. Jacoby D. J. Ozelius L. J. Ramesh V. Breakefield X. O. (2000). Mutant torsinA, responsible for early-onset torsion dystonia, forms membrane inclusions in cultured neural cells. Hum. Mol. Genet. 9, 1403–1413. 10.1093/hmg/9.9.1403 10814722

[b21] Holt C. E. Bullock S. L. (2009). Subcellular mRNA localization in animal cells and why it matters. Science 326, 1212–1216. 10.1126/science.1176488 19965463 PMC3785123

[b22] Jokhi V. Ashley J. Nunnari J. Noma A. Ito N. Wakabayashi-Ito N. Moore M. J. Budnik V. (2013). Torsin mediates primary envelopment of large ribonucleoprotein granules at the nuclear envelope. Cell Reports 3, 988–995. 10.1016/j.celrep.2013.03.015 23583177 PMC3683601

[b23] Jungwirth M. T. Kumar D. Jeong D. Y. Goodchild R. E. (2011). The nuclear envelope localization of DYT1 dystonia torsinA-ΔE requires the SUN1 LINC complex component. BMC Cell Biol. 12, 24 10.1186/1471-2121-12-24 21627841 PMC3164226

[b24] Kiebler M. A. Bassell G. J. (2006). Neuronal RNA granules: movers and makers. Neuron 51, 685–690. 10.1016/j.neuron.2006.08.021 16982415

[b25] Kiebler M. A. DesGroseillers L. (2000). Molecular insights into mRNA transport and local translation in the mammalian nervous system. Neuron 25, 19–28. 10.1016/S0896-6273(00)80868-5 10707969

[b26] Krishnakumar S. Burton D. Rasco J. Chen X. O'Donnell J. (2000). Functional interactions between GTP cyclohydrolase I and tyrosine hydroxylase in Drosophila. J. Neurogenet. 14, 1–23. 10.3109/01677060009083474 10938545

[b27] Kumer S. C. Vrana K. E. (1996). Intricate regulation of tyrosine hydroxylase activity and gene expression. J. Neurochem. 67, 443–462. 10.1046/j.1471-4159.1996.67020443.x 8764568

[b28] Mackay W. J. Reynolds E. R. O'Donnell J. M. (1985). Tissue-specific and complex complementation patterns in the Punch locus of Drosophila melanogaster. Genetics 111, 885–904. 3934035 10.1093/genetics/111.4.885 PMC1202678

[b29] Marck C. (1988). ‘DNA Strider’: a ‘C’ program for the fast analysis of DNA and protein sequences on the Apple Macintosh family of computers. Nucleic Acids Res. 16, 1829–1836. 10.1093/nar/16.5.1829 2832831 PMC338177

[b30] Maric M. Shao J. Ryan R. J. Wong C. S. Gonzalez-Alegre P. Roller R. J. (2011). A functional role for TorsinA in herpes simplex virus 1 nuclear egress. J. Virol. 85, 9667–9679. 10.1128/JVI.05314-11 21775450 PMC3196446

[b31] Medioni C. Mowry K. Besse F. (2012). Principles and roles of mRNA localization in animal development. Development 139, 3263–3276. 10.1242/dev.078626 22912410 PMC3424039

[b32] Neckameyer W. S. White K. (1993). Drosophila tyrosine hydroxylase is encoded by the pale locus. J. Neurogenet. 8, 189–199. 10.3109/01677069309083448 8100577

[b33] Nery F. C. Zeng J. Niland B. P. Hewett J. Farley J. Irimia D. Li Y. Wiche G. Sonnenberg A. Breakefield X. O. (2008). TorsinA binds the KASH domain of nesprins and participates in linkage between nuclear envelope and cytoskeleton. J. Cell Sci. 121, 3476–3486. 10.1242/jcs.029454 18827015 PMC3539201

[b34] Neuwald A. F. Aravind L. Spouge J. L. Koonin E. V. (1999). AAA+: A class of chaperone-like ATPases associated with the assembly, operation, and disassembly of protein complexes. Genomic Res. 9, 27–43. 9927482

[b35] O'Donnell J. M. McLean J. R. Reynolds E. R. (1989). Molecular and developmental genetics of the Punch locus, a pterin biosynthesis gene in Drosophila melanogaster. Dev. Genet. 10, 273–286. 10.1002/dvg.1020100316 2500290

[b36] Ozelius L. J. Page C. E. Klein C. Hewett J. W. Mineta M. Leung J. Shalish C. Bressman S. B. de Leon D. Brin M. F. (1999). The TOR1A (DYT1) gene family and its role in early onset torsion dystonia. Genomics 62, 377–384. 10.1006/geno.1999.6039 10644435

[b37] Sciamanna G. Bonsi P. Tassone A. Cuomo D. Tscherter A. Viscomi M. T. Martella G. Sharma N. Bernardi G. Standaert D. G. (2009). Impaired striatal D2 receptor function leads to enhanced GABA transmission in a mouse model of DYT1 dystonia. Neurobiol. Dis. 34, 133–145. 10.1016/j.nbd.2009.01.001 19187797 PMC3786200

[b38] Sciamanna G. Tassone A. Martella G. Mandolesi G. Puglisi F. Cuomo D. Madeo G. Ponterio G. Standaert D. G. Bonsi P. (2011). Developmental profile of the aberrant dopamine D2 receptor response in striatal cholinergic interneurons in DYT1 dystonia. PLoS ONE 6, e24261 10.1371/journal.pone.0024261 21912682 PMC3166312

[b39] Sciamanna G. Tassone A. Mandolesi G. Puglisi F. Ponterio G. Martella G. Madeo G. Bernardi G. Standaert D. G. Bonsi P. (2012). Cholinergic dysfunction alters synaptic integration between thalamostriatal and corticostriatal inputs in DYT1 dystonia. J. Neurosci. 32, 11991–12004. 10.1523/JNEUROSCI.0041-12.2012 22933784 PMC3471539

[b40] Segawa M. (2009). Autosomal dominant GTP cyclohydrolase I (AD GCH 1) deficiency (Segawa disease, dystonia 5; DYT 5). Chang Gung Med. J. 32, 1–11. 19292934

[b41] Song W. Onishi M. Jan L. Y. Jan Y. N. (2007). Peripheral multidendritic sensory neurons are necessary for rhythmic locomotion behavior in Drosophila larvae. Proc. Natl. Acad. Sci. USA 104, 5199–5204. 10.1073/pnas.0700895104 17360325 PMC1820883

[b42] St Johnston D. (2005). Moving messages: the intracellular localization of mRNAs. Nat. Rev. Mol. Cell Biol. 6, 363–375. 10.1038/nrm1643 15852043

[b43] Tarsy D. Simon D. K. (2006). Dystonia. N. Engl. J. Med. 355, 818–829. 10.1056/NEJMra055549 16928997

[b44] Thöny B. Auerbach G. Blau N. (2000). Tetrahydrobiopterin biosynthesis, regeneration and functions. Biochem. J. 347, 1–16. 10.1042/0264-6021:3470001 10727395 PMC1220924

[b45] Vale R. D. (2000). AAA proteins. Lords of the ring. J. Cell Biol. 150, F13–F20. 10.1083/jcb.150.1.F13 10893253 PMC2185557

[b46] Vasudevan A. Breakefield X. O. Bhide P. G. (2006). Developmental patterns of torsinA and torsinB expression. Brain Res. 1073-1074, 139–145. 10.1016/j.brainres.2005.12.087 16458269 PMC1472621

[b47] Wakabayashi-Ito N. Doherty O. M. Moriyama H. Breakefield X. O. Gusella J. F. O'Donnell J. M. Ito N. (2011). Dtorsin, the Drosophila ortholog of the early-onset dystonia TOR1A (DYT1), plays a novel role in dopamine metabolism. PLoS ONE 6, e26183 10.1371/journal.pone.0026183 22022556 PMC3192163

[b48] Willis D. K. Wang J. Lindholm J. R. Orth A. Goodman W. G. (2010). Microarray analysis of juvenile hormone response in Drosophila melanogaster S2 cells. J. Insect Sci. 10, 66 10.1673/031.010.6601 20672983 PMC3014815

[b49] Xiao J. Gong S. Zhao Y. LeDoux M. S. (2004). Developmental expression of rat torsinA transcript and protein. Brain Res. Dev. Brain Res. 152, 47–60. 10.1016/j.devbrainres.2004.05.012 15283994

[b50] Zhao C. Brown R. S. Chase A. R. Eisele M. R. Schlieker C. (2013). Regulation of Torsin ATPases by LAP1 and LULL1. Proc. Natl. Acad. Sci. USA 110, E1545–E1554. 10.1073/pnas.1300676110 23569223 PMC3637692

