# Supplementary material for: Mutant human torsinA, responsible for early-onset dystonia, dominantly suppresses GTPCH expression, dopamine levels and locomotion in Drosophila melanogaster
Source: Biol Open. 2015 Apr 17;4(5):585–95. doi: 10.1242/bio.201411080 (PMC4434810; doi:10.1242/bio.201411080)
Supplement: Supplementary Material [file supp_4_5_585__index.html]

Mutant human torsinA, responsible for early-onset dystonia, dominantly suppresses GTPCH expression, dopamine levels and locomotion in Drosophila melanogaster — Mutant human torsinA, responsible for early-onset dystonia, dominantly suppresses GTPCH expression, dopamine levels and locomotion in Drosophila melanogaster — Supplementary Material 

# Mutant human torsinA, responsible for early-onset dystonia, dominantly suppresses GTPCH expression, dopamine levels and locomotion in *Drosophila melanogaster*

## bio.201411080 Supplementary Material

**Files in this Data Supplement:**

- Supplementary Material - Noriko Wakabayashi-Ito et al. doi: 10.1242/bio.201411080
