# Supplementary material for: Mutant human torsinA, responsible for early-onset dystonia, dominantly suppresses GTPCH expression, dopamine levels and locomotion in Drosophila melanogaster
Source: Biol Open. 2015 Apr 17;4(5):585–95. doi: 10.1242/bio.201411080 (PMC4434810; doi:10.1242/bio.201411080)
Supplement: Supplementary Material [file supp_bio.201411080_bio.201411080-s1.pdf]

Biology Open (2015) 000, 1–11 doi:10.1242/bio.201411080

Supplementary Material

Noriko Wakabayashi-Ito et al. doi: 10.1242/bio.201411080

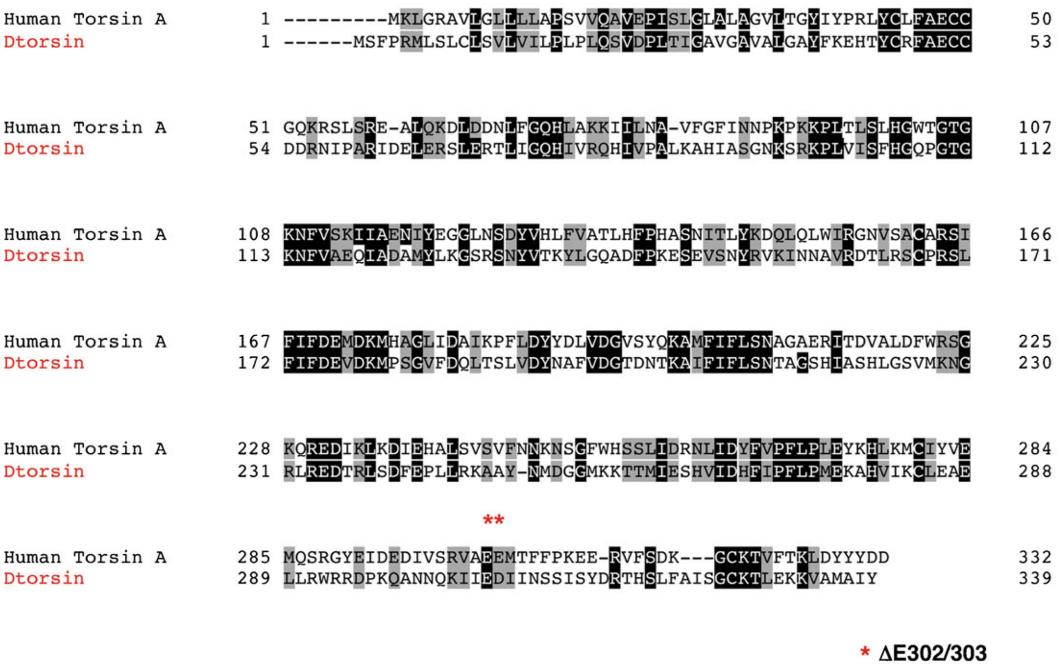

Fig. S1. Alignment of human torsinA and *Drosophila* Dtorsin protein sequences. Human torsinA (AAC51732) and *Drosophila dtorsin* (Torsin) (NP_572178) amino acid sequences were aligned using DNA strider (Marck, 1988) (version 1.4f19). Two possible locations of a single glutamate deletion (ΔE302 or ΔE303) are indicated by red asterisks. Identical amino acids are marked by black boxes. Conserved amino acids are marked by gray boxes.

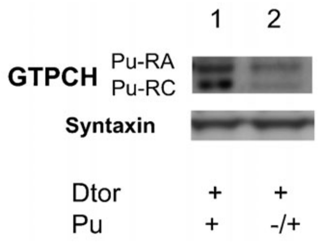

Fig. S2. GTPCH RA and RC forms are both decreased in *Pu*^{z22} heterozygous mutant. Adult head extracts were analyzed by western blots. The membrane was probed with rabbit anti-GTPCH A/C (upper panel) and reprobed with mouse anti-syntaxin (lower panel). The genotypes are: (1) Canton S-B (wild type) males, and (2) *Pu*^{Z22}/+ (*Pu* null mutation) males. The locations of GTPCH (Pu-RA: 45 kDa, Pu-RC: 43 kDa) and syntaxin (33 kDa) are indicated. Twenty µg of proteins were loaded in each lane.

Biology Open (2015) 000, 1–11 doi:10.1242/bio.201411080

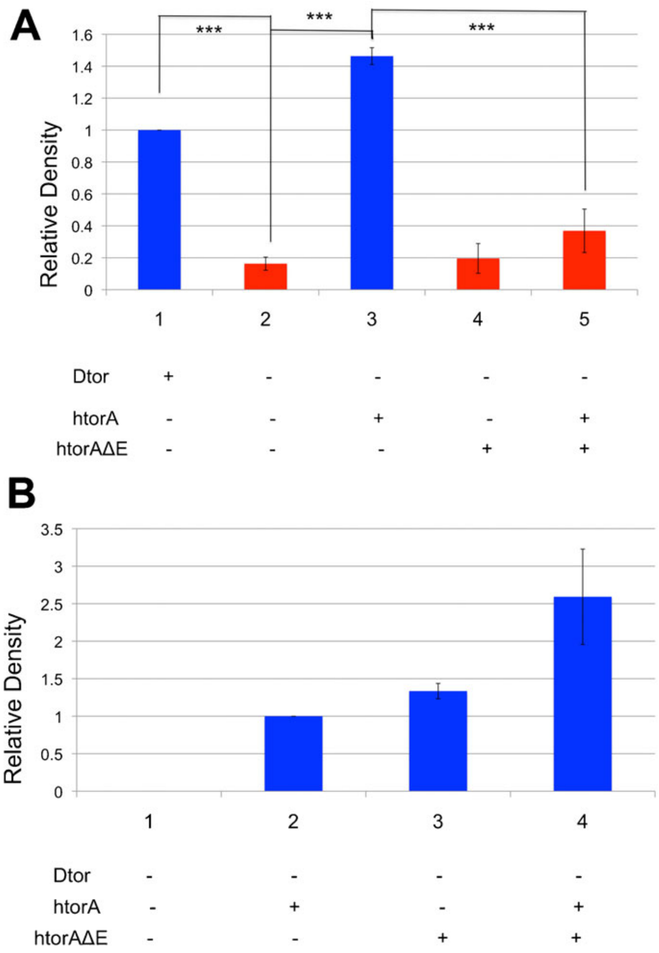

Fig. S3. Neuronal expression of human torsinAΔE has a dominant-negative effect on GTPCH protein levels in adult brains. (A) Adult head extracts were analyzed by western blots. The membrane was probed with rabbit anti-GTPCH A/C and reprobed with rabbit anti-actin. The relative densities of combined GTPCH bands (RA and RC) are calculated using actin bands as internal standards as shown here. The genotypes are: (1) *y w* (wild type) males (n=3), (2) *w elavGAL4 dtorsin*^{KO13}/Y (*dtorsin*-null) males (n=3), (3) *w elavGAL4 dtorsin*^{KO13}/Y; UAS-htorsinAΔE/+ males (n=3), (4) *w elavGAL4 dtorsin*^{KO13}/Y; UAS-htorsinA/+ males (n=3), (5) *w elavGAL4 dtorsin*^{KO13}/Y; UAS-htorsinA, UAS-htorsinAΔE/+ males (n=3). ***$p<0.0001$. (B) Adult head extracts were analyzed by western blots. The membrane was probed with rabbit anti-human torsinA and rabbit anti-actin antibodies. The relative densities of combined two human torsinA bands are calculated using actin bands as internal standards as shown here. The genotypes are: (1) *w elavGAL4 dtorsin*^{KO13}/Y (*dtorsin*-null) males (n=3), (2) *w elavGAL4 dtorsin*^{KO13}/Y; UAS-htorsinA/+ males (n=3), (3) *w elavGAL4 dtorsin*^{KO13}/Y; UAS-htorsinAΔE/+ males (n=3), (4) *w elavGAL4 dtorsin*^{KO13}/Y; UAS-htorsinA/+; UAS-htorsinAΔE/+ males (n=3). ***$p<0.0001$.

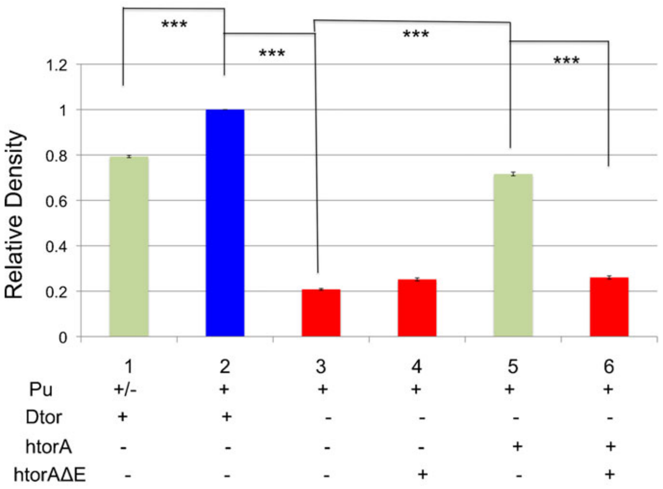

Fig. S4. Neuronal expression of human torsinAΔE has a dominant-negative effect on GTPCH protein levels in larval brains. Larval brain extracts were analyzed by western blots. The membrane was probed with rabbit anti-GTPCH A/C and reprobed with mouse anti-syntaxin. The relative densities of combined two human torsinA bands are calculated as syntaxin bands as internal standards as shown here. The genotypes are: (1) *Pu*^{Z22}/+ (*Pu* null mutation) males, (2) Canton S-B (wild type) males, (3) *y w dtorsin*^{KO13}/Y (*dtorsin*-null) males, (4) *w elavGAL4 dtorsin*^{KO13}/Y; UAS-htorsinAΔE/+ males, (5) *w elavGAL4 dtorsin*^{KO13}/Y; UAS-htorsinA/+ males, (6) *w elavGAL4 dtorsin*^{KO13}/Y; UAS-htorsinA, UAS-htorsinAΔE/+ males. ***$p<0.0001$.

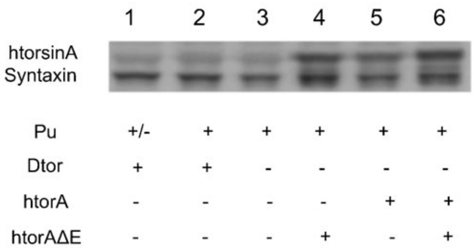

Fig. S5. Expression of human torsinA protein in larval brains driven by the pan-neuronal elavGAL4. (A) Larval brain extracts were analyzed by western blots. The membrane was probed with rabbit anti-human torsinA and mouse anti-syntaxin antibodies. The genotypes are: (1) *Pu*^{Z22}/+ (*Pu* null mutation) males, (2) Canton S-B (wild type) males, (3) *w elavGAL4 dtorsin*^{KO13}/Y (*dtorsin*-null) males, (4) *w elavGAL4 dtorsin*^{KO13}/Y; UAS-htorsinAΔE/+ males, (5) *w elavGAL4 dtorsin*^{KO13}/Y; UAS-htorsinA/+ males, (6) *w elavGAL4 dtorsin*^{KO13}/Y; UAS-htorsinA, UAS-htorsinAΔE/+ males. The locations of human torsinA and are indicated. Twenty μg of proteins were loaded in each lane.

Biology Open

 Biology Open (2015) 000, 1–11 doi:10.1242/bio.201411080

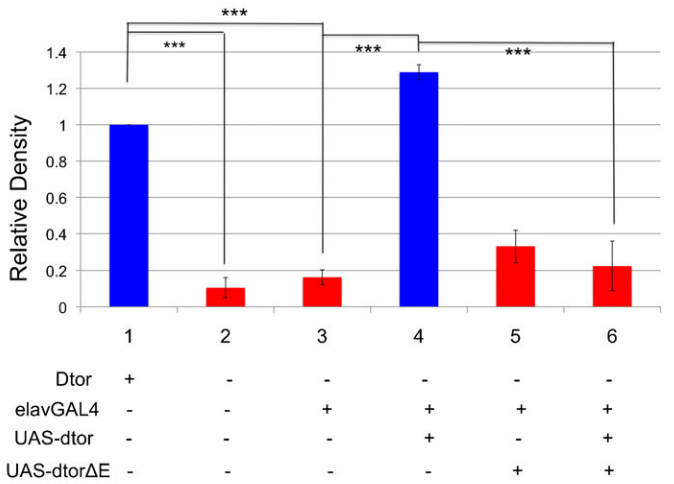

Fig. S6. Neuronal expression of *Drosophila* DtorsinΔE has a dominant-negative effect on GTPCH protein levels in adult brains. Adult head extracts were analyzed by western blots. The membrane was probed with rabbit anti-GTPCH A/C and reprobed with rabbit anti-actin. The relative densities of combined GTPCH bands (RA and RC) are calculated as actin bands as internal standards as shown here. The genotypes are: (1) *y w*/Y (wild type) males (n=3), (2) *y w dtorsin*^{KO13}/Y (*dtorsin*-null) males (n=3), (3) *w elavGAL4 dtorsin*^{KO13}/Y (*dtorsin*-null) males (n=3), (4) *w elavGAL4 dtorsin*^{KO13}/Y; UAS-*dtorsin*(B5)(II)/+ males (n=3), (5) *w elavGAL4 dtorsin*^{KO13}/Y; UAS- *dtorsinΔE*(#12)(III)/+ males (n=3), (6) *w elavGAL4 dtorsin*^{KO13}/Y; UAS-*dtorsin*(B5)(II): UAS-*dtorsinΔE*(#12)(III)/+ males (n=3). ***$p<0.0001$.

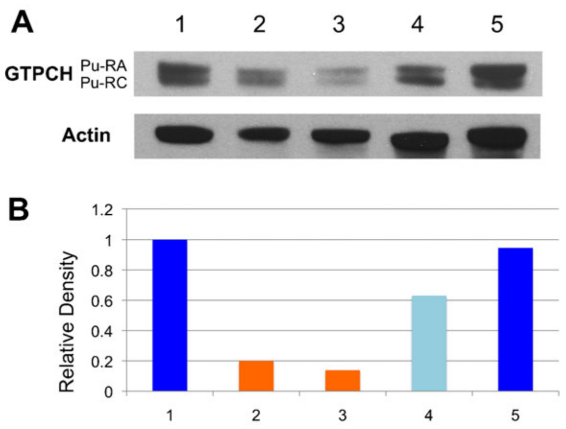

Fig. S7. Neuronal expression of *Drosophila* DtorsinΔD substantially rescues GTPCH protein levels in adult brains. (A) Adult head extracts were analyzed by western blots. The membrane was probed with rabbit anti-GTPCH A/C (upper panel) and reprobed with rabbit anti-actin (lower panel). The genotypes are: (1) *y w*/Y (wild type) males, (2) *w elavGAL4 dtorsin*^{KO13}/Y (*dtorsin*-null) males, (3) *w elavGAL4 dtorsin*^{KO13}/Y; UAS- *dtorsinΔE*/+ males, (4) *w elavGAL4 dtorsin*^{KO13}/Y; UAS- *dtorsinΔD*/+ males, (5) *w elavGAL4 dtorsin*^{KO13}/Y; UAS-*dtorsin*(B5)(II)/+ males. The locations of GTPCH (Pu-RA: 45 kDa, Pu-RC: 43 kDa) and actin (42 kDa) are indicated. Thirty µg of proteins were loaded in each lane. (B) The relative densities of combined GTPCH bands (RA and RC) are calculated as actin bands as internal standards as shown here. The genotypes are the same as in panel A of this figure.

Biology Open
